# *CYP2D6* Genotype and Tamoxifen Response for Breast Cancer: A Systematic Review and Meta-Analysis

**DOI:** 10.1371/journal.pone.0076648

**Published:** 2013-10-02

**Authors:** Danny W. K. Lum, Pablo Perel, Aroon D. Hingorani, Michael V. Holmes

**Affiliations:** 1 Centre for Cardiovascular Genetics, Institute of Cardiovascular Science, University College London, London, United Kingdom; 2 Faculty of Epidemiology and Public Health, London School of Hygiene and Tropical Medicine, London, United Kingdom; 3 Centre for Clinical Pharmacology, Division of Medicine, University College London, London, United Kingdom; 4 Genetic Epidemiology Group, Faculty of Population Health Sciences, Institute of Cardiovascular Science, University College London, London, United Kingdom; University of Modena & Reggio Emilia, Italy

## Abstract

**Objective:**

To evaluate evidence on the association between *CYP2D6* genotype and tamoxifen response through.

**Design:**

Systematic review and meta-analysis of prospective, cross-sectional and case-control studies published to 2012. For each study, relative risks and 95% confidence intervals were extracted and pooled with a fixed and random effects model. Heterogeneity, publication bias, subgroup, and meta-regression analyses were performed.

**Data Sources:**

PubMed (inception-2012) and EMBASE (inception-2012).

**Eligibility Criteria for Selecting Studies:**

Criteria for inclusion were studies reporting breast cancer outcomes in patients treated with tamoxifen and genotyped for polymorphisms in the *CYP2D6* gene.

**Results:**

Twenty-five studies of 13,629 individuals were identified, of which 22 investigated the association of *CYP2D6* genotype with outcomes in breast cancer women all receiving tamoxifen treatment (“treatment-only” design). Three randomized trials evaluated the effect of *CYP2D6* genotype on tamoxifen response (“effect modification” design). In analysis of treatment-only studies, the relative risk (RR) of all-cause mortality (>307 events in 4,936 patients) for carriers of a *CYP2D6* reduced function allele was 1.11 (95% confidence interval (CI): 0.94 to 1.31) compared to individuals with normal/increased function *CYP2D6* alleles. When we investigated a composite outcome including all-cause mortality and surrogate endpoints for overall survival (>307 events in 6,721 patients), carriers of a *CYP2D6* reduced function allele had a RR of 1.27 (95% CI: 1.11 to 1.45). From two randomized trials that permitted effect-modification analysis, one had only 154 patients and showed evidence of effect modification of tamoxifen by *CYP2D6* genotype for distant recurrence but was directionally opposite to that predicted, whereas a larger trial of 2,537 patients failed to show evidence of effect modification for breast cancer-free interval (P values for interaction 0.02 and 0.44, respectively).

**Conclusions:**

Based on these findings, there is insufficient evidence to recommend *CYP2D6* genotyping to guide tamoxifen treatment.

## Introduction

Breast cancer is one of the leading causes of cancer-related mortality in women [1,2]. Tamoxifen, a selective estrogen receptor modulator, was first approved by the U.S. Food and Drug Administration (FDA) in 1977 for the treatment of metastatic breast cancer [3], and has also been approved for primary prevention in women at high risk of breast cancer, for adjuvant treatment (given after primary treatment), and for ductal carcinoma *in situ* [4].

A meta-analysis of 20 randomized clinical trials (RCTs) including 10,645 women with estrogen receptor positive breast cancer demonstrated that, compared to no tamoxifen, adjuvant tamoxifen reduced breast cancer recurrence (relative risk (RR) 0.53, 95% CI: 0.47 to 0.59) and breast cancer mortality (RR 0.71, 95% CI: 0.61 to 0.81) at 5 years [5]. Furthermore, the benefit of tamoxifen persisted if treatment was continued for 10 years after its initiation [6].

Tamoxifen itself is only a weak modulator of the estrogen receptor while its metabolites (including endoxifen) are thought to be many times more potent [7]. Several enzymes are involved in tamoxifen metabolism (Figure **S1**) [8], and recent attention has focused on the hepatic cytochrome P450 2D6 (CYP2D6), with interest that genetic variants in *CYP2D6* that alter the level or activity of the encoded enzyme might alter the response to tamoxifen [9]. A U.S. Food and Drug Administration (FDA) Clinical Pharmacology Subcommittee convened in 2006 and agreed that “the scientific evidence on the metabolism of tamoxifen demonstrates CYP2D6 is an important pathway in the formation of endoxifen” and recommended the drug label be updated to reflect this [10].

We investigated the evidence of the association between *CYP2D6* genotype and response to tamoxifen treatment in individuals with breast cancer by conducting a systematic review and meta-analysis.

## Methods

To be eligible for inclusion, studies reported breast cancer outcomes in relation to *CYP2D6* genotype in humans of any ethnic group.

### Search strategy

Following PRISMA guidance [11], we searched PubMed and EMBASE from inception to 29^th^ January 2012. The search terms included the generic and proprietary drug names (including Novaldex, Zitazonium, Soltamox and Tomaxithen) and the gene name (*CYP2D6*, NCBI Entrez Gene 1565, and less specific search terms such as Cytochrome P450). Full details of the search string are provided in the Supplementary Methods reported in Text **S1**. We limited the search to studies that reported the association between *CYP2D6* genotype and tamoxifen response in humans. Eligible studies had an abstract containing the keywords “tamoxifen” and “*CYP2D6*” and reported original data. Editorials and reviews were omitted. The search was complemented by hand-searches of the bibliographies of included articles and of narrative reviews.

The search was conducted by DWKL and a random subset of studies was double-checked by MVH and ADH. Uncertainties were resolved by consensus. When questions remained, corresponding authors were contacted. The literature search was not restricted by ethnicity or language.

### Grouping of *CYP2D6* alleles

We used information from The Human Cytochrome P450 (CYP450) Allele Nomenclature Database (http://www.cypalleles.ki.se, Accessed 2013 Sep 4) and the Pharmacogenetics Knowledgebase (http://www.pharmgkb.org, Accessed 2013 Sep 4) to classify *CYP2D6* genotypes. For the main analysis, we compared individuals with any reduced/non-functional *CYP2D6* allele to individuals with normal/increased function alleles. In studies that reported individual *CYP2D6* genotype comparisons (e.g. *4 vs. *1), individuals carrying one or more reduced functional alleles (i.e. *9, *10, *17, *29, *36 and *41 alleles) and/or non-functional alleles (i.e. *3, *4, *5, *6, *7, *8, *11, *12, *13, *14, *15, *16, *19, *20, *21, *38, *40 and *42 alleles) were grouped together and compared with individuals carrying normal/increased function *CYP2D6* alleles (i.e. *1, *2, *33 and *35 alleles). For studies that grouped *CYP2D6* alleles into so-called “predicted phenotypes”, the poor metabolizers and intermediate metabolizers were grouped (as these approximate to carriage of one or more reduced functional alleles) and were compared with extensive metabolizers and ultra metabolizers (please see Table **S1** for further details). We expected individuals carrying the reduced function *CYP2D6* alleles to have higher risk of breast cancer outcomes (on the basis that less tamoxifen would be metabolized to the active metabolites in these individuals) compared to individuals that did not harbour these alleles.

In addition to the main genetic classification, we investigated a dose-response relationship by studying individuals with one copy vs. two copies of any reduced/non-functional *CYP2D6* allele compared to the normal/increased function alleles (please see Text **S1** for full details).

### Outcomes analysed in the meta-analysis

For the treatment-only studies (in which all individuals were exposed to tamoxifen), we synthesized a hierarchy to reflect clinical importance of the reported study outcomes based on recent narrative reviews of clinical trials in cancer [12–15]. We had three main outcomes of interest. First, our primary outcome was specific to mortality and termed “all-cause mortality”, which included breast cancer specific mortality, overall survival and all-cause mortality. Second, we included surrogate endpoints for overall survival such as progression free survival (PFS), which included non-fatal events. Thus, this secondary outcome was a composite of all-cause mortality and surrogate endpoints for overall survival. Third, to maximise the number of studies that could be incorporated into the analysis, we added outcomes that consisted of non-fatal events (such as any recurrence, metastases, breast cancer free interval) to the composite. This third outcome therefore consisted of a composite of all-cause mortality, surrogate endpoints for overall survival (including non-fatal events), and non-fatal events. For each of these three main outcomes, we used a hierarchy such that if a study reported fatal and non-fatal events separately, only the fatal events from this study would contribute towards each of the three composites. Further details of the three composite outcomes and the contribution of individual studies to each outcome are provided in Figure **S2** and Table **S2**.

In addition to the main outcomes, we conducted separate meta-analyses for the following individual outcomes for the treatment-only analysis: (i) breast cancer-specific mortality; (ii) overall survival/all-cause mortality; (iii) surrogate endpoints for overall survival (including non-fatal events); (iv) non-fatal events (breast cancer recurrence), and; (v) the adverse drug reaction, hot flushes.

For the effect modification studies, we used outcomes reported in the clinical trials.

We extracted counts of events by genotype and used these to generate odds ratios for each study. When event counts were not available, we used the reported univariate effect estimates. When neither event counts nor univariate estimates were reported, we used the reported multivariate effect estimates. When a study reported both single *CYP2D6* genotype (e.g. *4 vs. *1) and grouped *CYP2D6* genotypes comparisons (e.g. poor metabolizers vs. extensive metabolizers), the latter was used as it incorporated more genetic variation in *CYP2D6*.

### Statistical analysis

The analysis was split into two main types depending on the design [16]: the treatment-only meta-analysis was limited to studies of individuals all receiving tamoxifen, and the effect-modification analysis was based on an evaluation of heterogeneity in the tamoxifen treatment effect size in groups of differing genotypes studied within randomised controlled trials.

For the treatment-only analysis, we used fixed and random effects meta-analysis models to pool estimates from individual studies. Unless otherwise stated, meta-analyses used the fixed effects model [17]. The summary relative risk (RR, incorporating odds ratio, hazards ratio and rate ratio) was used to describe the pooled summary estimates derived from meta-analyses. Heterogeneity between studies was quantified using the *I* ^2^ statistic and tested using Cochrane’s Q test [17].

For the effect-modification analysis, we used the Bland and Altman method [18] to test for evidence of heterogeneity between subgroups of the clinical trials, grouped according to *CYP2D6* genotype.

We used several techniques to investigate potential sources of bias. Publication bias was investigated by visual inspection of the funnel plot and quantified by the intercept from linear regression [19]. The stability of each summary effect estimate was evaluated using an influence analysis that investigated the effects of removing each individual study from the meta-analysis on the overall meta-analysis summary. We conducted subgroup analyses of the composite all-cause mortality and surrogate endpoints for overall survival outcome to investigate if the association differed by study design, patient characteristics, treatment and genotyping method (see Text **S1** for full details). We used this outcome for subgroup analyses as the sample size was larger. Finally, we investigated risk of bias in the randomized trials used for effect modification analysis using criteria from the Cochrane Handbook for clinical trials [17].

We investigated the potential for a dose-response relationship on the treatment-only analysis by conducting meta-regression analyses, testing a linear relationship between the number of *CYP2D6* reduced function alleles and the risk of each composite outcome.

All statistical analyses were performed using Stata v11.2 (StataCorp, College Station, Texas, USA) and reported P-values are two-sided. For the treatment-only analysis, a P-value <0.05 was used to indicate evidence against the null hypothesis of no association. In the subgroup analysis of treatment-only studies (see Text **S1**), we adjusted the P-value (using the Bonferroni method) to take into account multiple testing, thus our threshold for nominal significance on subgroup analysis was P<0.002 (calculated from 0.05/23).

## Results

### Study inclusion

We identified 25 studies including 13,629 participants that fulfilled our selection criteria (Figure **S3**). The characteristics of the 25 studies are presented in Table **1** and Tables **S3**-**S5**.

**Table 1:** Characteristics of the 25 studies identified by the systematic review.

First author, reference, year	Study design	Total partici-pants	Duration of follow-up (years)	Ethnicity	Age (years)	Breast cancer stage	Estrogen receptor (ER) status	Progest-erone receptor (PR) status	Outcomes reported
Abraham et al. [37], 2010	Cohort	3155	NR	Caucasian (98.8%)	53	I, II, III, IV or unknown	ER+, ER- or unknown	NR	BCSS, OS/all-cause mortality
Bijl et al. [38], 2009	Cohort	85	NR	Caucasian	75.5	NR	NR	NR	Breast cancer mortality, Cancer mortality, All-cause mortality
Goetz et al. [39], 2005	Cohort	223	11.4	Caucasian (92%)	68	NR	ER+	NR	OS, DFS, RFT, Hot flush
Gonzalez et al. [40], 2007	Cohort	84	5.5	NR	51.5	I, II or III	ER+ or ER-	PR+ or PR-	Disease recurrence/ relapse
Kiyotani et al. [41], 2011	Cohort	462	6.8	Asian	51	NR	ER+, ER- or unknown	PR+, PR- or unknown	RecFS
Lammers et al. [42], 2010	Cohort	99	NR	Caucasian (95.1%), Asian (3.9%), African (1%)	51.8	NR	ER+	PR+	OS, TTP
Lash et al. [43], 2011	Case-Control	1682	NR	Caucasian	NR	I, II or III	ER+, ER- or unknown	NR	Breast cancer recurrence
Madlensky et al. [44], 2009	Cohort	1411	NR	NR	NR	I or II	ER+	NR	Hot flush
Morrow et al. [45], 2011	Case-Control	106	9	NR	58	0/I, II or III	ER+ or ER-	PR+ or PR-	Disease recurrence
Newman et al. [46], 2008	Cohort	205	NR	Caucasian (7.8% Ashkenazi Jewish)	43	NR	ER+ or ER-	NR	OS, RecFS, TTRec
Nowell et al. [47], 2005	Cohort	337	5.4	Caucasian (81%), African–American (19%).	NR	I, II, III or IV	ER+ or ER-	PR+ or PR-	OS, PFS
Okishiro et al. [48], 2009	Cohort	173	4.9	Asian	47	NR	ER+ or ER-	PR+ or PR-	RecFS
Park et al. [49], 2011	Cohort	110	6.2	Asian	43.6	NR	ER+, ER- or unknown	PR+, PR- or unknown	RecFS, OS
Park et al. [50], 2011	Cohort	716	5.6	Asian	45	I, II, or III	ER+, ER- or unknown	PR+, PR- or unknown	RecFS
Rae et al. [20], 2012	RCT	1203	10	Caucasian	NR	I, II, or IIIA	ER+, ER- or unknown	PR+, PR- or unknown	Distant recurrence rate, Any recurrence rate
Regan et al. [22], 2012	RCT	2193	6	Caucasian (98%)	61	NR	ER+ or ER-	PR+	BCFI, Hot flush
Schroth et al. [51], 2007	Cohort	486	5.9	Caucasian	60	NR	ER+ or ER-	NR	OS, EFS, RFT, Relapse risk
Schroth et al. [52], 2009	Cohort	1325	6.3	Caucasian	66	I, II or III	ER+, ER- or unknown	PR+, PR- or unknown	OS, DFS, EFS, TTRec
Stingl et al. [53], 2010	Cohort	493	7	Caucasian	59	NR	ER+	NR	Disease related event recurrence, TTP, PFS
Teh et al. [54], 2011	Cohort	95	NR	Asian	51	0-II or III&IV	ER+ or ER-	PR+ or PR-	Recurrence & metastasis risk
Thompson et al. [55], 2010	Cohort	618	4.9 (Cohort 1), 9.4 (Cohort 2)	Caucasian	60.5 (Cohort 1), 63.1 (Cohort 2)	I, II or III	ER+	NR	RFS
van Schaik et al. [56], 2011	Cohort	742	NR	Caucasian (>95%)	59	NR	ER+	PR+, PR- or unknown	TTF
Wegman et al. [21], 2005	RCT	226	10.7	Caucasian	NR	NR	ER+ or ER-	NR	Distant recurrence rate, Distant RFS
Wegman et al. [57], 2007	Cohort	677	7.1	Caucasian	69	II or III	ER+	NR	RecFS
Xu et al. [58], 2008	Cohort	293	5.3	Asian	NR	0, I, II, III or unknown	ER+, ER- or unknown	PR+, PR- or unknown	DSS, DFS

**Footnotes**: BCFI: breast cancer-free interval, BCSS: breast cancer-specific survival, Cohort 1: Dundee, UK, Cohort 2: Manchester, UK, DFS: disease-free survival, DSS: disease-specific survival, EFS: event-free survival, ER- estrogen receptor negative, ER+: estrogen receptor positive, HR: hazard ratio, NA: not applicable, NR: not recorded, OR: odds ratio, OS: overall survival, PFS: progression-free survival, PR- progesterone receptor negative, PR+: progesterone receptor positive, RCT: randomized-controlled trial, RFS: relapse-free survival, RFT: relapse-free time, RecFS: recurrence-free survival, TAM- tamoxifen non-treated group, TAM+: tamoxifen-treated group, TTF: time to-treatment failure, TTP: time to progression, TTRec: Time to recurrence. Age represents mean age at diagnosis, first tamoxifen use or surgery.

All identified studies were conducted in women. The median age was 58 years (range 41 to 76) and median sample size per study was 462 participants (range 84 to 3155). In the 17 of 25 (68%) studies that reported duration of follow-up, the median duration was 6.3 years (range 4.5 to 11.4). Most studies (16 of 22 that reported ethnicity) reported data principally on Caucasian individuals. The breast cancer stage of study participants ranged from 0 to IV (details reported in Table **1**). Twenty-four of 25 (96%) studies reported information on hormone receptor status, with the majority of studies (17 of 24, 71%) evaluating a mixture of ER+ and ER- breast cancers. Only six of 25 (24%) studies reported that genotype ascertainment was conducted with blinding to clinical outcomes, and only three studies (12%) reported that clinical outcomes were ascertained with blinding to *CYP2D6* genotype (Table **S3**).

There were 21 unique outcomes reported across the 25 studies (Figure S4), which we grouped into three composite outcomes (Figure S2).

Thirty-six *CYP2D6* star (*) alleles were genotyped across the studies, with only four alleles (*4, *5, *6, *41) genotyped in half or more of the 13,629 individuals across the 25 studies. The most commonly genotyped *CYP2D6* alleles were *4 in 23 studies (genotyped in 96% of participants), followed by *41 in 13 studies (genotyped in 77% of participants). There were several * alleles that were genotyped but not reported (Figure S5).

### Treatment-only meta-analysis of composite outcomes

#### All-cause mortality

**Figure 1:**
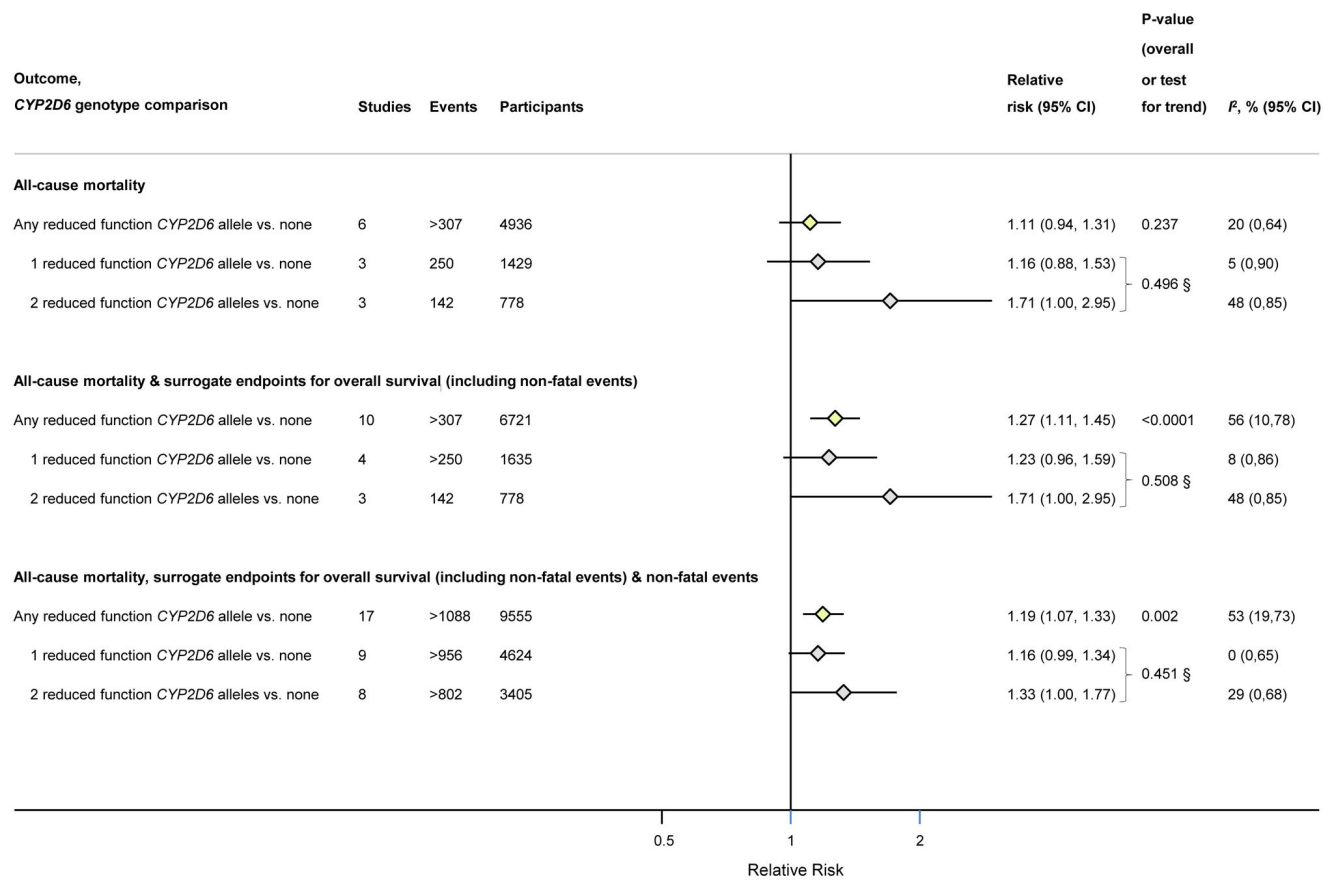
Meta-analysis pooled estimates from treatment-only analysis of the association of any, one or two copies of a reduced function *CYP2D6* allele vs. none with composite breast cancer outcomes. Footnotes: § P-value represents test for trend, conducted using metaregression assuming a linear dose-response relationship between number of *CYP2D6* alleles and summary effect estimate.

Six studies (>307 events in 4,936 participants) reported data that could be incorporated into the all-cause mortality analysis. Compared to those with normal or increased function alleles, individuals with any copy of a reduced function *CYP2D6* allele had a RR of death of 1.11 (95% CI: 0.94 to 1.31; *I* ^2^=20%) in the fixed effects analysis and 1.12 (95% CI: 0.90 to 1.41) in random effects meta-analysis (Figure **1**, Table **S6** and Figure **S6**).

#### All-cause mortality and surrogate endpoints for overall survival

When we studied a composite encompassing all-cause mortality as well as surrogate endpoints for overall survival, a total of 10 studies (>307 events in 6,721 participants) could be incorporated into the analysis. Individuals with any copy of a reduced function *CYP2D6* allele had a RR of achieving the composite of 1.27 (95% CI: 1.11 to 1.45; *I* ^2^=56%) for fixed effects and 1.34 (95% CI: 1.06 to 1.69) for random effects meta-analysis compared to individuals with normal/increased function *CYP2D6* alleles (Figure **1**, Table **S6** and Figure **S7**).

#### All-cause mortality, surrogate endpoints for overall survival and non-fatal outcomes

When our composite included all-cause mortality, surrogate endpoints for overall survival as well as non-fatal breast cancer events (17 studies, >1,088 events in 9,555 participants), individuals with any copy of a reduced function *CYP2D6* allele had a RR of achieving this end point of 1.19 (95% CI: 1.07 to 1.33; *I* ^2^=53%) for fixed effects and 1.22 (95% CI: 1.01 to 1.46) for random effects meta-analysis compared to those with normal or increased function *CYP2D6* alleles (Figure **1**, Table **S6** and Figure **S8**).

### Treatment-only meta-analysis of individual outcomes

**Figure 2:**
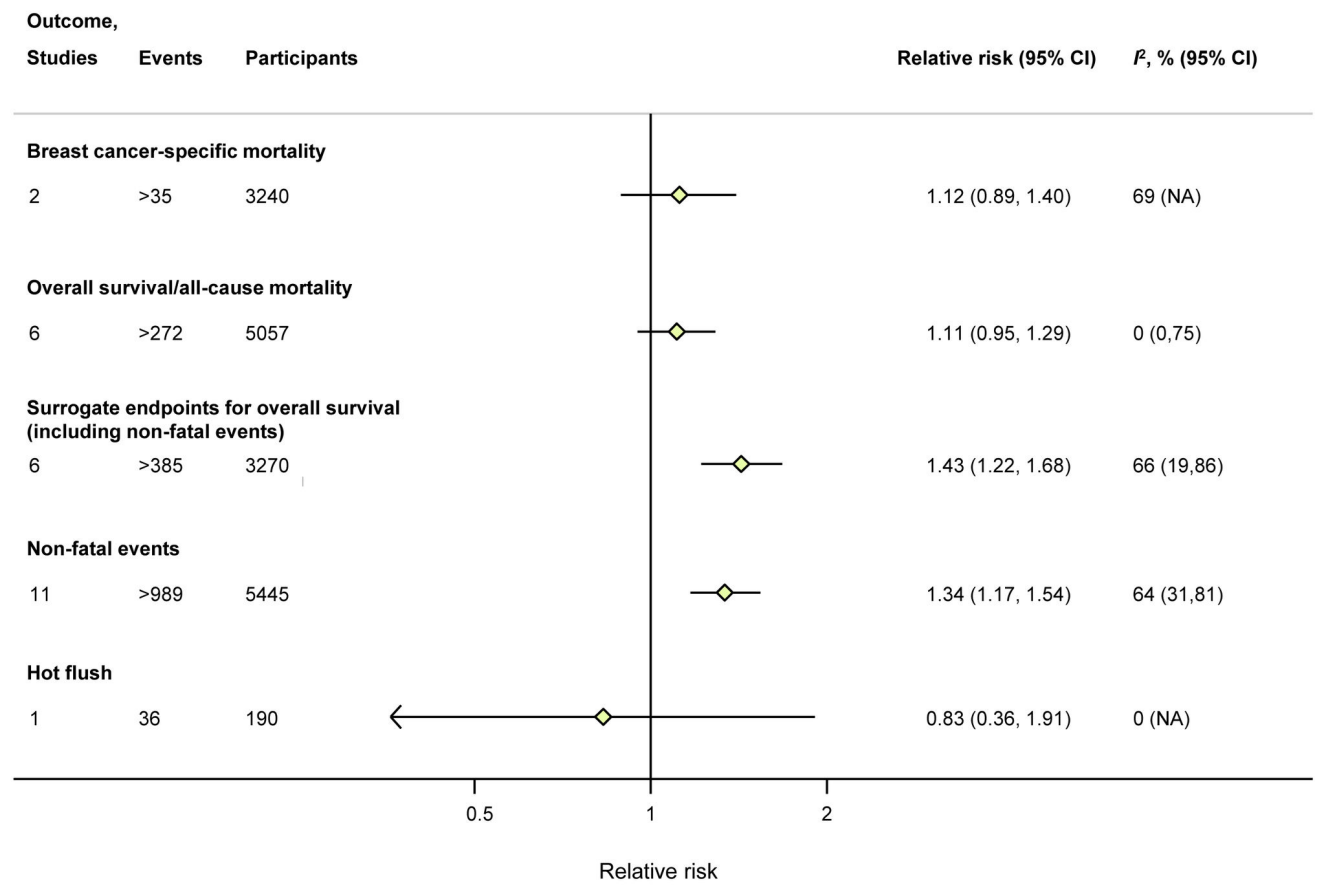
Meta-analysis pooled estimates from treatment-only analysis of the association of any *CYP2D6* reduced function alleles vs. none with individual outcomes. **Footnotes**: Non-fatal events included breast cancer outcomes that were not fatal.

In the treatment only analysis of individual outcomes, the risk for breast cancer-specific mortality (>35 events in 2 studies with 3,240 individuals) was RR 1.12; 95% CI: 0.89 to 1.40 and for overall survival/all-cause mortality (>272 events in 6 studies with 5,057 individuals) it was RR 1.11; 95% CI: 0.95 to 1.29) (Figure **2** and Table **S6**). Carriage of any copy of a reduced function *CYP2D6* allele was associated with meeting a surrogate endpoint for overall survival (>385 events in 6 studies with 3,270 individuals; RR 1.43; 95% CI: 1.22 to 1.68) as well as non-fatal recurrence (>989 events in 11 studies with 5,445 individuals; RR 1.34; 95% CI: 1.17 to 1.54) (Figure **2** and Table **S6**).

### Subgroup analyses

**Figure 3:**
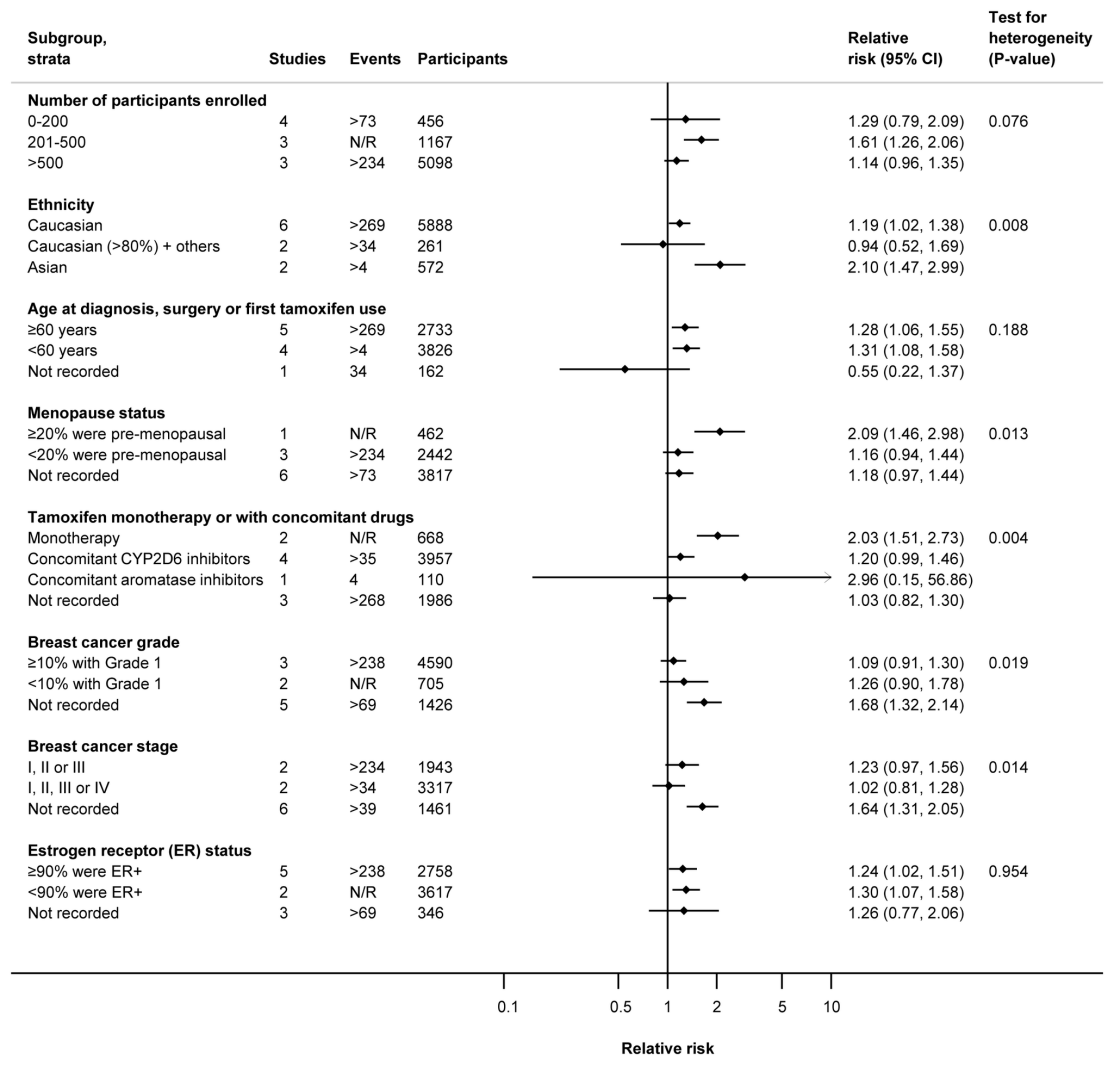
Subgroup analyses of the association between any reduced function *CYP2D6* allele vs. none for the composite outcome of all-cause mortality and surrogate endpoints for overall survival (including non-fatal events).

We conducted subgroup analysis for the composite that included all-cause mortality together with surrogate endpoints for overall survival (including non-fatal events). Although there was a suggestion of a differential association of *CYP2D6* genotype according to patient ethnicity, concomitant therapy, breast cancer stage/grade and menopause status, none of the subgroup analyses surpassed our *a priori* Bonferroni-adjusted P-value threshold of <0.002 (Figure 3 and Figure S9).

### Publication bias and stability of effect estimate

The funnel plots for the three composite outcomes appeared symmetrical (Figure **S10**), supported by the lack of evidence on formal statistical testing (Harbord test P-values all >0.05). We did not identify evidence of excessive influence by any individual study for each of the three composite outcomes (Figure **S11**).

### Gene dose-response relationship

When we investigated the potential for a gene dose-response relationship between the number of reduced function *CYP2D6* alleles and composites outcomes in patients treated with tamoxifen, the point estimate for two reduced function *CYP2D6* alleles was consistently of greater magnitude than for one copy in each of the three composite outcomes, however the 95% CI were wide and there was no evidence for a trend on formal statistical testing (all P values >0.05) (Figure **1**).

### Effect-modification analysis

Of 25 potentially eligible studies, three were trials with participants randomized to tamoxifen or a comparator group (Table **1**). One of the trials [20] did not report information in a way that permitted analysis of heterogeneity of treatment effect by genotype category. Of the two remaining trials, the risk of bias for one trial was high, and for the other it was low (Table **S7**).

In the trial reported by Wegman et al. [21] of 154 patients (in which the risk of bias was high, Table **S7**), the OR of distant recurrence for individuals with any copy of the CYP2D6*4 reduced function allele was 0.18 (95% CI: 0.05 to 0.61) for tamoxifen compared to no tamoxifen, whereas for individuals with the normal *CYP2D6* allele (*1/*1), the corresponding OR was 0.96 (95% CI: 0.45 to 2.04). A test for interaction yielded evidence for effect modification (P=0.02) (Table **2**). However, the point estimate for the CYP2D6*4 allele was directionally opposite to that expected according to the predicted phenotypic consequence of the genetic variant.

**Table 2:** Trials reporting *CYP2D6* genotype as an effect modifier of tamoxifen response.

Author	Tam-oxifen dose	Duration of follow-up	Comparator arm	Outcome	*CYP2D6* genotype/ predicted phenotype	No. of individuals randomized	No. of clinical events	OR	95% CI	Interaction P-value §
Regan et al. [22], 2012	20 mg/day	Median: 6 years	Letrozole	**Breast cancer-free interval**	**reduced function** [PM (*3,*4,*6 or *7/*3,*4,*6 or *7) and IM (*41/*41,*3,*4,*6 or *7/*41, or wt/*3,*4,*6,*7 or *41)]	952	126	1.05	0.72-1.52	0.44
					**normal function** [EM (*1/*1)]	1585	207	1.26	0.94-1.69	
				**Hot flush**	**reduced function** [PM (*3,*4,*6 or *7/*3,*4,*6 or *7) and IM (*41/*41,*3,*4,*6 or *7/*41, or wt/*3,*4,*6,*7 or *41)]	1659	714	1.31	1.08-1.59	0.52
					**normal function** [EM (*1/*1)]	2734	1064	1.20	1.03-1.40	
Wegman et al. [21], 2005	40 mg/day	Mean: 10.7 years	No tamoxifen	**Distant recurrence**	**reduced function** (*4)	47	21	0.18	0.05-0.61	0.02
					**normal function** (*1/*1)	107	52	0.96	0.45-2.04	

**Footnotes**: CI: confidence interval, EM: extensive metabolizer, IM: intermediate metabolizer, OR: odds ratio, PM: poor metabolizer, RCT: randomized clinical trial, *wt*: wild-type. §P-value for interaction obtained from Bland and Altman method [18].

In the trial reported by Regan et al. [22] including 2,537 participants randomized to tamoxifen or the aromatase inhibitor letrozole, two outcomes were analysed: (i) breast cancer-free interval (defined from recruitment to the first breast cancer event [local, regional, or distant recurrence] or a new invasive contralateral breast cancer) and (ii) hot flushes. The OR for breast cancer-free interval for tamoxifen compared to letrozole in individuals with any reduced function *CYP2D6* allele was 1.05 (95% CI: 0.72 to 1.52), and for individuals with normal function *CYP2D6* allele, it was 1.26 (95% CI: 0.94 to 1.69), with no evidence for effect modification (P=0.44) (Table **2**). There was also no evidence for an interaction between *CYP2D6* genotype and tamoxifen therapy for the adverse outcome of hot flush (P=0.52) (Table **2**).

It was not possible to provide a pooled summary estimate for the effect of tamoxifen versus comparator group stratified by *CYP2D6* genotype, as the comparator groups used and the outcomes reported were different in the two trials.

## Discussion

We investigated the evidence base on the association between *CYP2D6* genotype and clinical outcomes following tamoxifen treatment for breast cancer. A total of 25 studies were identified comprising 13,629 patients with breast cancer adding to prior meta-analyses [23,24] by including 5 more studies, providing new insight on a range of outcomes, investigating a potential gene dose-response relationship, and an assessment of data from trials that investigated effect modification of tamoxifen by *CYP2D6* genotype status.

In the treatment-only analysis for all-cause mortality, the point estimate was consistent in direction with that expected from the hypothesis that reduced tamoxifen metabolism is associated with a poorer outcome but the confidence limits were wide and included the null value (of RR=1). When we used a less stringent composite outcome encompassing all-cause mortality and surrogate endpoints for overall survival (including non-fatal events), individuals carrying any copy of a reduced function *CYP2D6* allele had a higher risk of an adverse outcome than individuals with normal/increased function *CYP2D6* alleles. In the analysis of the individual outcomes, we did not identify an association between *CYP2D6* genotype and mortality, but did find an association between *CYP2D6* genotype and surrogate endpoints for overall survival (including non-fatal events). Although it was not possible to pool information from the treatment trials in the effect-modification analysis, because of the different comparator arms, neither trial individually provided support for the hypothesis that carriage of reduced function *CYP2D6* alleles modified the treatment response to tamoxifen, regardless of whether the comparator was a placebo or letrozole (a drug that is not considered to be metabolized by CYP2D6) [25]. However, as is common with many randomized trials, power may be a real limitation of subgroup analyses [26]. Taken together, these data provide inconsistent and inconclusive evidence on the clinical implications of individual variation in CYP2D6 metaboliser status and the outcome from tamoxifen treatment.

The association of *CYP2D6* genotype with surrogate endpoints for overall survival (both individually and as part of a composite) on the treatment-only analysis is worthy of discussion. Controversy exists on the validity of using surrogates when evaluating cancer treatments [27]. For decades, overall survival has been considered the optimal endpoint of investigation for assessing treatment efficacy in medical oncology, partly due to the simplicity of case ascertainment [12]. However, use of overall survival requires a large sample size and long duration of follow-up, increasing the cost of trials in which it is the primary outcome. This has fuelled debate about whether it is possible to use surrogate endpoints to make inferences regarding a treatment’s efficacy on overall survival [28–32]. Importantly, the FDA has recently approved drugs based on these surrogate measures [33]. Criticisms of the use of surrogate endpoints for overall survival are that they may be prone to investigator bias and sensitive to the time of assessment, making them potentially unsuitable for cohort studies and non-blinded randomized trials [34] and that they may not reflect clinically-meaningful outcomes [14].

This systematic review and meta-analysis has several limitations that are worthy of comment. First, the lack of trials available for the effect modification analysis and differences in the treatment regimens and outcomes reported in the trials meant that we could not conduct a meta-analysis of effect modification analyses. However, the largest trial provided no evidence for effect modification. Second, we used aggregate rather than participant data, which meant we were limited in the precision with which we could conduct subgroup analyses and our ability to homogenize the genotype comparisons or outcomes reported. In an attempt to address this, we used a genetic analysis model (any reduced function *CYP2D6* allele versus none) that aimed to simplify the genetic comparison, which allowed us to include all studies in the analysis. Although the outcomes reported did differ considerably between studies, our approach was to generate composite outcomes to pool the individual outcomes reported across studies to maximize power. Because use of composites may obscure associations with individual constituents, we also investigated the association of *CYP2D6* genotype with individual outcomes.

Our systematic review is timely and an important addition to recent findings from two large clinical trials [20,22] that both failed to show evidence of a *CYP2D6* genotype by tamoxifen interaction, and the subsequent debate that ensued [9,35,36]. Furthermore, this systematic review and meta-analysis is likely to add to the on-going debate on the potential use of surrogate markers for studies of breast cancer outcomes. In the absence of a validated surrogate marker for overall survival (the gold-standard outcome, for which we did not identify an association of *CYP2D6* genotype on either the composite all-cause mortality or individual outcome analyses), the associations between *CYP2D6* genotype and surrogate endpoints for overall survival that we identified should be interpreted with caution.

## Conclusions

Despite a weak association between *CYP2D6* genotype and surrogate endpoints for overall survival, we did not identify an association between *CYP2D6* genotype and tamoxifen response for all-cause mortality or overall survival. The current evidence does not support the use of *CYP2D6* genotyping to guide tamoxifen prescribing for the treatment of breast cancer.

## Supporting Information

Text S1 **Supplementary Methods.**
(PDF)

Table S1 **Nomenclature for the CYP2D6 phenotype predicted from *CYP2D6* alleles.**
(PDF)

Table S2 **Contribution of included studies from the systematic review to each of the composite outcomes.**
(PDF)

Table S3 **Clinical and genotype characteristics of the 25 studies included in the systematic review.**
(PDF)

Table S4 **Participant, genotype and treatment characteristics of the 25 studies included in the systematic review.**
(PDF)

Table S5 **Breast cancer characteristics of the 25 studies included in the systematic review.**
(PDF)

Table S6 **Meta-analysis pooled estimates for the composite and individual outcomes comparing fixed and random effects models.**
(PDF)

Table S7 **Risk of bias in the two randomized trials that conducted an effect modification analysis.**
(PDF)

Figure S1 **Metabolism of tamoxifen by the hepatic cytochrome P450 (CYP) enzymes and the mechanism of the endoxifen (4-hydroxy-N-desmethyl-tamoxifen) metabolite in breast cell growth inhibition.**
(PDF)

Figure S2 **Generation of the composite outcomes from outcomes reported in the studies identified from the systematic review.**
(PDF)

Figure S3 **PRISMA flow diagram for systematic review of the association of CYP2D6 genotype and tamoxifen response in breast cancer patients.**
(PDF)

Figure S4 **Unique outcomes reported by studies and the grouping into main outcomes for analyses.**
(PDF)

Figure S5 Proportions of 13,629 individuals in the 25 studies that were genotyped for the 36 *CYP2D6* * alleles.
(PDF)

Figure S6 **Meta-analysis (fixed and random effects models) of the association of any reduced function *CYP2D6* allele versus none for the risk of all-cause mortality.**
(PDF)

Figure S7 **Meta-analysis (fixed and random effects models) of the association of any reduced function *CYP2D6* allele versus none with the composite outcome of all-cause mortality and surrogate endpoints for overall survival (including non-fatal events).**
(PDF)

Figure S8 **Meta-analysis (fixed and random effects models) of the association of any reduced function *CYP2D6* allele versus none with the composite outcome of all-cause mortality, surrogate endpoints for overall survival (including non-fatal events) and non-fatal events.**
(PDF)

Figure S9 **Subgroup analysis of the association of any reduced function *CYP2D6* allele versus none for the composite outcome of all-cause mortality and surrogate endpoints for overall survival (including non-fatal events).**
(PDF)

Figure S10 **Funnel plot of any reduced function *CYP2D6* allele versus none for the composite outcomes.**
(PDF)

Figure S11 **Influence analysis of any reduced function *CYP2D6* allele versus none for the composite outcomes.**
(PDF)

Checklist S1 **PRISMA checklist.**
(DOC)
