# Supplementary material for: CYP2D6 Genotype and Tamoxifen Response for Breast Cancer: A Systematic Review and Meta-Analysis
Source: PLoS One. 2013 Oct 2;8(10):e76648. doi: 10.1371/journal.pone.0076648 (PMC3788742; doi:10.1371/journal.pone.0076648)
Supplement: Text S1 — Supplementary Methods. (PDF) [file pone.0076648.s001.pdf]

Text S1: Supplementary Methods

Search criterion

PubMed search string

PubMed MeSH search
(1) "tamoxifen"[MeSH Terms]; (2) "cytochromes"[MeSH Terms]; (3) "cytochrome p 450 enzyme system"[MeSH Terms]; (4) "hydroxylation"[MeSH Terms]; (5) "imipramine"[MeSH Terms]; (6) "sparteine"[MeSH Terms]; (7) "oxygenases"[MeSH Terms]; (8) "cell proliferation"[MeSH Terms]; (9) 2 OR 3 OR 4 OR 5 OR 6 OR 7 OR 8; (10) "genotype"[MeSH Terms]; (11) "genes"[MeSH Terms]; (12) "genetic association studies"[MeSH Terms]; (13) "alleles"[MeSH Terms]; (14) "polymorphism, genetic"[MeSH Terms]; (15) "polymorphism, single nucleotide"[MeSH Terms]; (16) 10 OR 11 OR 12 OR 13 OR 14 OR 15; (17) 1 AND 9 AND 16

PubMed text word search
(1) tamoxifen OR ICI-46,474 OR ICI-46474 OR ICI-47699 OR Nolvadex OR Novaldex OR Soltamox OR Tamoxifen Citrate OR Tomaxithen OR Zitazonium; (2) "Valodex"; (3) "Istubal"; (4) N-desmethyl tamoxifen; (5) 4-hydroxytamoxifen; (6) endoxifen; (7) 1 OR 2 OR 3 OR 4 OR 5 OR 6; (8) Cytochrome*; (9) Cytochrome-P450*; (10) Cytochrome P450; (11) Cytochrome P-450 OR Cytochrome P-450 Monooxygenase OR Cytochrome P-450 Oxygenase OR Cytochrome P-450-Dependent Monooxygenase; (12) CYP2D6*; (13) Cytochrome P450 2D6 OR Debrisoquine 4-Hydroxylase OR Debrisoquine 4-Monooxygenase OR Debrisoquine Hydroxylase OR Imipramine 2-Hydroxylase OR Sparteine Monooxygenase; (14) 8 OR 9 OR 10 OR 11 OR 12 OR 13; (15) Genotyp*; (16) Gene; (17) Allele*; (18) Polymorphism*; (19) 15 OR 16 OR 17 OR 18; (20) 7 AND 14 AND 19

EMBASE search string

EMBASE subject heading search
(1) exp tamoxifen/; (2) exp tamoxifen citrate/; (3) 1 OR 2; (4) exp cytochrome/; (5) exp cytochrome P450/; (6) exp oxygenase/; (7) exp cytochrome P450 2D/; (8) exp cytochrome P450 2D6/; (9) exp debrisoquine 4 hydroxylase/; (10) exp 2 hydroxyimipramine/; (11) exp 4' hydroxytamoxifen/; (12) 4 OR 5 OR 6 OR 7 OR 8 OR 9 OR 10 OR 11; (13) exp genotype/; (14) exp genotype phenotype correlation/; (15) exp gene/; (16) exp genetic association/; (17) exp allele/; (18) exp genetic polymorphism/; (19) 13 OR 14 OR 15 OR 16 OR 17 OR 18; (20) 3 AND 12 AND 19

EMBASE text word search
(1) (tamoxifen or ICI-46,474 or ICI-46474 or ICI-47699 or Nolvadex or Novaldex or Soltamox or Tamoxifen Citrate or Tomaxithen or Zitazonium).af.; (2) Valodex.mp.; (3) Istubal.mp.; (4) N-desmethyl tamoxifen.mp.; (5) 4-

hydroxytamoxifen.mp.; (6) endoxifen.mp.; (7) 1 or 2 or 3 or 4 or 5 or 6; (8) Cytochrome$.mp.; (9) Cytochrome-P450$.mp.; (10) Cytochrome P450.mp.; (11) (Cytochrome P-450 or Cytochrome P-450 Monooxygenase or Cytochrome P-450 Oxygenase or Cytochrome P-450-Dependent Monooxygenase).af.; (12) CYP2D6$.mp.; (13) (Cytochrome P450 2D6 or Debrisoquine 4-Hydroxylase or Debrisoquine 4-Monooxygenase or Debrisoquine Hydroxylase or Imipramine 2-Hydroxylase or Sparteine Monooxygenase).af.; (14) 8 or 9 or 10 or 11 or 12 or 13; (15) Genotyp$.mp.; (16) Gene$.mp.; (17) Allele$.mp.; (18) Polymorphism$.mp.; (19) 15 or 16 or 17 or 18; (20) 7 and 14 and 19

In addition, the full text articles of two randomized clinical trials (the ATAC and the BIG 1-98 clinical trials) that initially presented their results in abstract form at the 2010 San Antonio Breast Cancer Symposium (SABCS) were included.[1,2]

Grouping of *CYP2D6* alleles for the investigation of dose-response relationship (one or two versus no reduced function *CYP2D6* alleles)

For the 'one reduced function *CYP2D6* allele versus none' comparison, studies that reported individuals with one copy of a reduced function or non-functional *CYP2D6* allele were compared to the reference wildtype (**1/*1*). The corresponding categorisation for the predicted phenotype studies was IM status (including heterozygous extensive metabolizer [hetEM] and heterozygous intermediate metabolizer [hetIM]) versus EM/UM phenotype. For the 'two reduced function *CYP2D6* alleles versus none' comparison, studies that reported individuals with two copies of a reduced function and/or non-functional allele were compared to the reference wild-type (**1/*1*). The corresponding categorisation for the predicted phenotype studies was PM status versus EM/UM phenotype.

Subgroup analyses

Subgroups were defined based on the following baseline clinical and methodological characteristics:

i. Study characteristics comprised of design [case-control, cohort, randomized-controlled trial (RCT)], number of enrolled participants, number of clinical events, duration of follow-up, loss to follow-up, genotype ascertainment with outcome blinded, outcome ascertainment with genotype blinded, and matching features (for case-control studies only).

ii. Participant characteristics comprised of ethnicity, age and menopause status (pre-, peri- or post-menopause).

iii. Genotype characteristics comprised of genotype platform, genotype call rate, whether the *CYP2D6* alleles genotyped were in Hardy-Weinberg equilibrium (HWE), and the genotypes (whether single or grouped) compared.

iv. Treatment characteristics comprised of concordance to tamoxifen, and whether tamoxifen was administered as a monotherapy or given with concomitant drugs.

v. Breast cancer characteristics comprised of breast cancer type (primary, metastatic or others), grade (1, 2 or 3) and stage (I, II, III or IV), lymph node involvement, tumour size, estrogen receptor (ER) and progesterone receptor (PR) statuses.

vi. Reported data characteristics comprised of type of data (raw event counts, hazard ratio, odds ratio or rate ratios) and whether the summary estimates (if any) were adjusted for clinical and/or other factors.

We used the Chi-square statistic from the '`metan`' command in Stata 11.2 (StataCorp, College Station, Texas, USA) to test for differences between subgroups.

References

1. Rae JM, Drury S, Hayes DF, Stearns V, Thibert JN, et al. (2010) [S1-7] Lack of correlation between gene variants in tamoxifen metabolizing enymes with primary endpoints in the ATAC trial. In: University of Michigan, Ann Arbor; University of Michigan; Royal Marsden Hospital, United Kingdom; Breakthrough Breast Cancer Research Centre, United Kingdom; Johns Hopkins University; CRUK Centre for Epidemiology, Mathematics and Statistics, United Kingdom. 33rd Annual San Antonio Breast Cancer Symposium (SABCS) San Antonio, Texas, USA, 8-12 December 2010. Available at: http://www.abstracts2view.com/sabcs10/view.php?nu=SABCS10L_1093 [Accessed 29 January 2012].

2. Leyland-Jones B, Regan MM, Bouzyk M, Kammler R, Tang W, et al. (2010) [S1-8] Outcome according to CYP2D6 genotype among postmenopausal women with endocrine-responsive early invasive breast cancer randomized in the BIG 1-98 trial. In: BIG 1-98 Collaborative Group and International Breast Cancer Study Group, Bern, Switzerland. 33rd Annual San Antonio Breast Cancer Symposium (SABCS) San Antonio, Texas, USA, 8-12 December 2010. Available at: http://www.abstracts2view.com/sabcs10/view.php?nu=SABCS10L_556 [Accessed 29 January 2012].
