# Supplementary material for: CYP2D6 Genotype and Tamoxifen Response for Breast Cancer: A Systematic Review and Meta-Analysis
Source: PLoS One. 2013 Oct 2;8(10):e76648. doi: 10.1371/journal.pone.0076648 (PMC3788742; doi:10.1371/journal.pone.0076648)
Supplement: Table S1 — Nomenclature for the CYP2D6 phenotype predicted from CYP2D6 alleles. (PDF) [file pone.0076648.s002.pdf]

Table S1: Nomenclature for the CYP2D6 phenotype predicted from *CYP2D6* alleles.

***CYP2D6* alleles**	**Expected effect on CYP2D6 enzymatic function (predicted phenotype)**
2 copies of any of the following non-functional alleles: **3, *4, *5, *6, *7, *8, *11, *12, *13, *14, *15, *16, *19, *20, *21, *38, *40, *42*	Poor metabolizer (PM)
2 copies of any of the following reduced function alleles: **9, *10, *17, *29, *36, *41*,<br><br>OR<br><br>1 copy of any of the following non-functional alleles: **3, *4, *5, *6, *7, *8, *11, *12, *13, *14, *15, *16, *19, *20, *21, *38, *40, *42*, AND<br>1 copy of any of the following functional alleles: **1, *2, *33, *35*,<br><br>OR<br><br>1 copy of any of the following non-functional alleles: **3, *4, *5, *6, *7, *8, *11, *12, *13, *14, *15, *16, *19, *20, *21, *38, *40, *42*, AND<br>1 copy of any of the following reduced function alleles: **9, *10, *17, *29, *36, *41*	Intermediate metabolizer (IM)
2 copies of any of the following functional alleles: **1, *2, *33, *35*	Extensive metabolizer (EM)
More than 2 copies of any of the following functional alleles: **1, *2, *33, *35*	Ultra metabolizer (UM)
