# Supplementary material for: CYP2D6 Genotype and Tamoxifen Response for Breast Cancer: A Systematic Review and Meta-Analysis
Source: PLoS One. 2013 Oct 2;8(10):e76648. doi: 10.1371/journal.pone.0076648 (PMC3788742; doi:10.1371/journal.pone.0076648)
Supplement: Table S5 — Breast cancer characteristics of the 25 studies included in the systematic review. (PDF) [file pone.0076648.s006.pdf]

Table S5: Breast cancer characteristics of the 25 studies included in the systematic review.

Author	BC type	BC grade	Lymph node (N) involvement	Tumour (T) size
**Abraham *et al.*,[1] 2010**	Invasive	1, 2, 3 or unknown	N0, N1, N2, N3 or unknown	T1, T2 or T3
**Bijl *et al.*,[2] 2009**	NR	NR	NR	NR
**Goetz *et al.*,[3] 2005**	NR	NR	N0, N1, N2, or N3	<3cm or >3cm
**Gonzalez *et al.*,[4] 2007**	NR	NR	Negative (N0) or positive	NR
**Kiyotani *et al.*,[5] 2011**	Primary invasive	NR	Negative (N0), positive or unknown	T1, T2, T3 or unknown
**Lammers *et al.*,[6] 2010**	Metastatic	NR	NR	NR
**Lash *et al.*,[7] 2011**	NR	1, 2, 3 or unknown	NR	NR
**Madlensky *et al.*,[8] 2009**	Early stage	NR	NR	NR
**Morrow *et al.*,[9] 2011**	Early stage	NR	N0 or N1	Tis/T1, T2, or T3/T4
**Newman *et al.*,[10] 2008**	Familial early-onset	2 or 3	N0, N1, or ≥N2	<3cm or >3cm
**Nowell *et al.*,[11] 2005**	Primary invasive	1, 2 or 3	Negative (N0) or positive	T1, T2 or >3cm
**Okishiro *et al.*,[12] 2009**	Primary	1, 2 or 3	Positive or negative	T1 or T2
**Park *et al.*,[13] 2011**	Primary	1, 2 or 3	Negative (N0) or positive	T0, Tis, T1 or T2
**Park *et al.*,[14] 2011**	Early stage	NR	N0, N1, N2, N3 or unknown	T1, T2, T3, T4 or unknown
**Rae *et al.*,[15] 2012**	Invasive early-stage	1, 2, 3 or unknown	Negative (N0), positive or unknown	T1, T2 or T3
**Regan *et al.*,[16] 2012**	Early invasive	1, 2 or 3	Negative (N0) or positive	T1 or T2
**Schroth *et al.*,[17] 2007**	Primary invasive	1, 2 or 3	N0, N1 or N2	T1, T2 or T3
**Schroth *et al.*,[18] 2009**	NR	1, 2, 3 or unknown	N0, N1, N2+N3 or unknown	T1, T2 or unknown
**Stingl *et al.*,[19] 2010**	Incident	1, 2, or 3&4	Negative (N0) or positive	≤T1 or ≥T2
**Teh *et al.*,[20] 2011**	Invasive	1 or 2+3	NR	NR
**Thompson *et al.*,[21] 2010**	Invasive	NR	N0, N1, ≥N2 or unknown.	T1, T2, T3 or unknown
**van Schaik *et al.*,[22] 2011**	Invasive	1, 2, 3 or unknown	Negative (N0), positive or unknown	T1, T2 or unknown
**Wegman *et al.*,[23] 2005**	Unilateral	NR	Negative (N0) or positive	T1, T2 or >3cm
**Wegman *et al.*,[24] 2007**	NR	NR	N0, N1 or ≥N2	T1, T2 or T3
**Xu *et al.*,[25] 2008**	Primary early-stage	NR	Negative (N0) or positive	T1, T2 or unknown

Footnotes: BC: breast cancer; NR: not recorded; N0: negative lymph node involvement, N1, N2 & N3: positive lymph node involvement.

References

1. Abraham JE, Maranian MJ, Driver KE, Platte R, Kalmyrzaev B, et al. (2010) CYP2D6 gene variants: Association with breast cancer specific survival in a cohort of breast cancer patients from the United Kingdom treated with adjuvant tamoxifen. Breast Cancer Research 12.

2. Bijl MJ, van Schaik RH, Lammers LA, Hofman A, Vulto AG, et al. (2009) The CYP2D6*4 polymorphism affects breast cancer survival in tamoxifen users. Breast Cancer Res Treat 118: 125-130.

3. Goetz MP, Rae JM, Suman VJ, Safgren SL, Ames MM, et al. (2005) Pharmacogenetics of tamoxifen biotransformation is associated with clinical outcomes of efficacy and hot flashes. Journal of Clinical Oncology 23: 9312-9318.

4. Gonzalez-Santiago S, Zárate R, Haba-Rodríguez J, Gómez A, Bandrés E, et al. (2007) CYP2D6*4 polymorphism as blood predictive biomarker of breast cancer relapse in patients receiving adjuvant tamoxifen. *Journal of Clinical Oncology*, 2007 American Society of Clinical Oncology (ASCO) Annual Meeting Proceedings Part I. Vol 25, No. 18S (June 20 Supplement), 2007: 590.

5. Kiyotani K, Mushiroda T, Tsunoda T, Morizono T, Hosono N, et al. (2012) A genome-wide association study identifies locus at 10q22 associated with clinical outcomes of adjuvant tamoxifen therapy for breast cancer patients in Japanese. Hum Mol Genet.

6. Lammers LA, Mathijssen RHJ, Van Gelder T, Bijl MJ, De Graan AJM, et al. (2010) The impact of CYP2D6-predicted phenotype on tamoxifen treatment outcome in patients with metastatic breast cancer. British Journal of Cancer 103: 765-771.

7. Lash TL, Cronin-Fenton D, Ahern TP, Rosenberg CL, Lunetta KL, et al. (2011) CYP2D6 inhibition and breast cancer recurrence in a population-based study in Denmark. Journal of the National Cancer Institute 103: 489-500.

8. Madlensky L, Flatt SW, Natarajan L, Lawrence H, Nikoloff D, et al. (2009) Hot flashes are associated with CYP2D6 genotype in breast cancer survivors taking tamoxifen. Cancer Research Conference: 31st Annual San Antonio Breast Cancer Symposium San Antonio, TX United States Conference Start 69.

9. Morrow PK, Serna R, Broglio K, Pusztai L, Nikoloff DM, et al. (2011) Effect of CYP2D6 polymorphisms on breast cancer recurrence. Cancer.

10. Newman WG, Hadfield KD, Latif A, Roberts SA, Shenton A, et al. (2008) Impaired tamoxifen metabolism reduces survival in familial breast cancer patients. Clinical Cancer Research 14: 5913-5918.

11. Nowell SA, Ahn J, Rae JM, Scheys JO, Trovato A, et al. (2005) Association of genetic variation in tamoxifen-metabolizing enzymes with overall survival and recurrence of disease in breast cancer patients. Breast Cancer Research and Treatment 91: 249-258.

12. Okishiro M, Taguchi T, Kim SJ, Shimazu K, Tamaki Y, et al. (2009) Genetic polymorphisms of CYP2D6*10 and CYP2C19*2,*3 are not associated with prognosis, endometrial thickness, or bone mineral density in Japanese breast cancer patients treated with adjuvant tamoxifen. Cancer 115: 952-961.

13. Park HS, Choi JY, Lee MJ, Park S, Yeo CW, et al. (2011) Association between genetic polymorphisms of CYP2D6 and outcomes in breast cancer patients with tamoxifen treatment. J Korean Med Sci 26: 1007-1013.

14. Park IH, Ro J, Park S, Lim HS, Lee KS, et al. (2012) Lack of any association between functionally significant CYP2D6 polymorphisms and clinical outcomes in early breast cancer patients receiving adjuvant tamoxifen treatment. Breast Cancer Res Treat 131: 455-461.

15. Rae JM, Drury S, Hayes DF, Stearns V, Thibert JN, et al. (2012) CYP2D6 and UGT2B7 Genotype and Risk of Recurrence in Tamoxifen-Treated Breast Cancer Patients. Journal of the National Cancer Institute 104: 452-460.

16. Regan MM, Leyland-Jones B, Bouzyk M, Pagani O, Tang W, et al. (2012) CYP2D6 Genotype and Tamoxifen Response in Postmenopausal Women with Endocrine-Responsive Breast Cancer: The Breast International Group 1-98 Trial. Journal of the National Cancer Institute 104: 441-451.

17. Schroth W, Antoniadou L, Fritz P, Schwab M, Muerdter T, et al. (2007) Breast cancer treatment outcome with adjuvant tamoxifen relative to patient CYP2D6 and CYP2C19 genotypes. Journal of Clinical Oncology 25: 5187-5193.

18. Schroth W, Goetz MP, Hamann U, Fasching PA, Schmidt M, et al. (2009) Association between CYP2D6 polymorphisms and outcomes among women with early stage breast cancer treated with tamoxifen. JAMA - Journal of the American Medical Association 302: 1429-1436.

19. Stingl JC, Parmar S, Huber-Wechselberger A, Kainz A, Renner W, et al. (2010) Impact of CYP2D6*4 genotype on progression free survival in tamoxifen breast cancer treatment. Curr Med Res Opin 26: 2535-2542.

20. Teh LK, Mohamed NI, Salleh MZ, Rohaizak M, Shahrun NS, et al. (2011) The Risk of Recurrence in Breast Cancer Patients Treated with Tamoxifen: Polymorphisms of CYP2D6 and ABCB1. AAPS J.

21. Thompson AM, Johnson A, Quinlan P, Hillman G, Fontecha M, et al. (2011) Comprehensive CYP2D6 genotype and adherence affect outcome in breast cancer patients treated with tamoxifen monotherapy. Breast Cancer Research and Treatment 125: 279-287.
22. Van Schaik RHN, Kok M, Sweep FCJ, Van Vliet M, Van Fessem M, et al. (2011) The CYP2C19*2 genotype predicts tamoxifen treatment outcome in advanced breast cancer patients. Pharmacogenomics 12: 1137-1146.
23. Wegman P, Vainikka L, Stal O, Nordenskjold B, Skoog L, et al. (2005) Genotype of metabolic enzymes and the benefit of tamoxifen in postmenopausal breast cancer patients. Breast cancer research : BCR 7: R284-290.
24. Wegman P, Elingarami S, Carstensen J, Stal O, Nordenskjold B, et al. (2007) Genetic variants of CYP3A5, CYP2D6, SULT1A1, UGT2B15 and tamoxifen response in postmenopausal patients with breast cancer. Breast cancer research : BCR 9: R7.
25. Xu Y, Sun Y, Yao L, Shi L, Wu Y, et al. (2008) Association between CYP2D6 *10 genotype and survival of breast cancer patients receiving tamoxifen treatment. Annals of Oncology 19: 1423-1429.
