# Supplementary material for: CYP2D6 Genotype and Tamoxifen Response for Breast Cancer: A Systematic Review and Meta-Analysis
Source: PLoS One. 2013 Oct 2;8(10):e76648. doi: 10.1371/journal.pone.0076648 (PMC3788742; doi:10.1371/journal.pone.0076648)
Supplement: Table S6 — Meta-analysis pooled estimates for the composite and individual outcomes comparing fixed and random effects models. (PDF) [file pone.0076648.s007.pdf]

Table S6: Meta-analysis pooled estimates for the composite and individual outcomes comparing fixed and random effects models.

Outcomes	Reduced function *CYP2D6* allele §	No. of studies	Events	Participants	Fixed effects RR	95% CI	Random effects RR	95% CI	I^2	Cochrane's Q P-value
**COMPOSITE OUTCOMES**										
**All-cause mortality**	Any vs. none	6	>307	4936	1.11	0.94-1.31	1.12	0.90-1.41	19.6%	0.286
	1 vs. none	3	250	1429	1.16	0.88-1.53	1.19	0.85-1.68	5.4%	0.348
	2 vs. none	3	142	778	1.71	1.00-2.95	3.23	0.73-14.21	47.5%	0.149
**All-cause mortality & surrogate endpoints for overall survival (including non-fatal events)**	Any vs. none	10	>307	6721	1.27	1.11-1.45	1.34	1.06-1.69	55.8%	0.016
	1 vs. none	4	>250	1635	1.23	0.96-1.59	1.28	0.95-1.71	7.8%	0.354
	2 vs. none	3	142	778	1.71	1.00-2.95	3.23	0.73-14.21	47.5%	0.149
**All-cause mortality, surrogate endpoints for overall survival (including non-fatal events) & non-fatal outcomes**	Any vs. none	17	>1088	9555	1.19	1.07-1.33	1.22	1.01-1.46	53.4%	0.005
	1 vs. none	9	>956	4624	1.16	0.99-1.34	1.16	0.99-1.34	0.0%	0.477
	2 vs. none	8	>802	3405	1.33	1.00-1.77	1.36	0.93-1.97	29.4%	0.194
**INDIVIDUAL OUTCOMES**										
**Breast-cancer specific mortality**	Any vs. none	2	>35	3240	1.12	0.89-1.40	1.44	0.65-3.20	68.7%	0.074
**Overall survival/ All-cause mortality**	Any vs. none	6	>272	5057	1.11	0.95-1.29	1.11	0.95-1.29	0.0%	0.442
**Surrogate endpoints for overall survival (including non-fatal events)**	Any vs. none	6	>385	3270	1.43	1.22-1.68	1.37	1.01-1.85	66.3%	0.011
**Non-fatal outcomes**	Any vs. none	11	>989	5445	1.34	1.17-1.54	1.32	1.02-1.71	63.9%	0.002
**Hot flush**	Any vs. none	1	36	190	0.83	0.36-1.91	0.83	0.36-1.91	0.0%	NA

Footnotes: CI: confidence interval, I^2: I-squared heterogeneity statistic, NA: not applicable, RR: relative risk. § represents genotype comparison used for analysis
