# Supplementary material for: CYP2D6 Genotype and Tamoxifen Response for Breast Cancer: A Systematic Review and Meta-Analysis
Source: PLoS One. 2013 Oct 2;8(10):e76648. doi: 10.1371/journal.pone.0076648 (PMC3788742; doi:10.1371/journal.pone.0076648)
Supplement: Table S7 — Risk of bias in the two randomized trials that conducted an effect modification analysis. (PDF) [file pone.0076648.s008.pdf]

Table S7: Risk of bias in the two randomized trials that conducted an effect modification analysis.

First Author, Reference, Year	Random sequence generation	Allocation concealment	Blinding of participants and personnel	Blinding of outcome assessment	Incomplete outcome data	Selective reporting	Other bias	Overall risk of bias
**Regan *et al.***,[1] **2012**	Low (Minimization [restricted (permuted blocks) + stratification by study centre and therapy])	Low (not reported in trial, but other sources confirm)	Low (double-blinded)	Low (outcome assessors blinded)	Low (Loss to follow-up disclosed)	Low (additional endpoints reported but no outcomes unreported)	Low	**Low**
**Wegman *et al.***,[2] **2005**	Unclear (balanced lists)	Low (clinicians unaware of allocation until after patient identifiers recorded centrally)	High (no placebo, not stated if patients or trial personnel blinded)	High (not stated)	Low (Loss to follow-up disclosed)	Low (all outcomes reported)	Low	**High**

Footnotes: Bias assessed using Cochrane Handbook for Systematic Reviews of Interventions.

References

1. Regan MM, Leyland-Jones B, Bouzyk M, Pagani O, Tang W, et al. (2012) CYP2D6 Genotype and Tamoxifen Response in Postmenopausal Women with Endocrine-Responsive Breast Cancer: The Breast International Group 1-98 Trial. Journal of the National Cancer Institute 104: 441-451.
2. Wegman P, Vainikka L, Stal O, Nordenskjold B, Skoog L, et al. (2005) Genotype of metabolic enzymes and the benefit of tamoxifen in postmenopausal breast cancer patients. Breast cancer research : BCR 7: R284-290.
