# Supplementary material for: CYP2D6 Genotype and Tamoxifen Response for Breast Cancer: A Systematic Review and Meta-Analysis
Source: PLoS One. 2013 Oct 2;8(10):e76648. doi: 10.1371/journal.pone.0076648 (PMC3788742; doi:10.1371/journal.pone.0076648)
Supplement: Figure S1 — Metabolism of tamoxifen by the hepatic cytochrome P450 (CYP) enzymes and the mechanism of the endoxifen (4-hydroxy-N-desmethyl-tamoxifen) metabolite in breast cell growth inhibition. (PDF) [file pone.0076648.s009.pdf]

Figure S1: Metabolism of tamoxifen by the hepatic cytochrome P450 (CYP) enzymes and the mechanism of the endoxifen (4-hydroxy-*N*-desmethyl-tamoxifen) metabolite in breast cell growth inhibition.

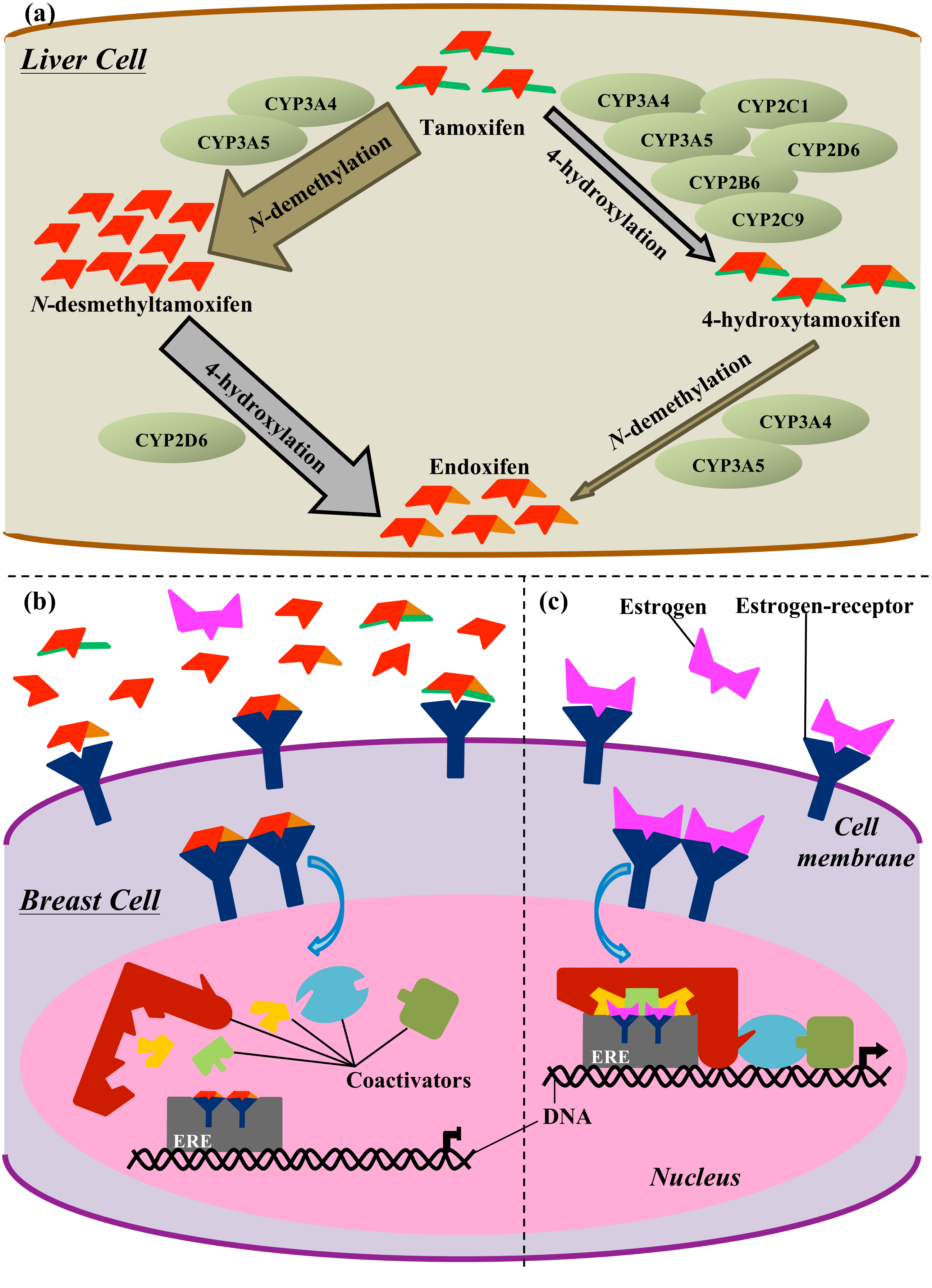

Footnotes: (a) Tamoxifen is metabolised by two pathways to endoxifen. The main metabolic pathway (>90% of tamoxifen metabolism, illustrated by a thicker arrow) involves *N*-demethylation (illustrated by brown arrows) by CYP3A4 and CYP3A5, which yields the more potent metabolite *N*-desmethyltamoxifen. *N*-desmethyltamoxifen then undergoes 4-hydroxylation (illustrated by grey arrows) by CYP2D6 to the more potent metabolite endoxifen (4-hydroxy-*N*-desmethyl-tamoxifen). The second (minor) pathway of tamoxifen metabolism (illustrated by a thinner arrow) involves 4-hydroxylation by multiple CYP isoforms including CYP2D6 to the metabolite 4-hydroxytamoxifen, which has shown to be 30- to 100-fold more potent than tamoxifen.[1] A further *N*-demethylation of 4-hydroxytamoxifen catalysed by CYP3A4 and CYP3A5 contributes a small percentage of plasma endoxifen concentration. Although endoxifen and 4-hydroxytamoxifen have comparable anti-estrogenic potency, plasma endoxifen concentration has shown to be much higher (6- to 10-fold) than that of 4-hydroxytamoxifen.[1,2] Endoxifen also has a 100-fold greater affinity for the estrogen receptor (ER) than tamoxifen or *N*-desmethyltamoxifen.[2] (b) The presence of endoxifen out-competes estrogen (as well as tamoxifen and *N*-desmethyltamoxifen) in binding to the ER at the breast cell membrane. The successful endoxifen-bound ERs form ER-dimer, which is then translocated into the nucleus (not drawn to scale). Endoxifen-bound ER-dimer then binds to the estrogen response element (ERE) and blocks recruitment of transcriptional coactivators, causing transcriptional repression and inhibition to breast cell growth. (c) In the absence of tamoxifen therapy, the estrogen-bound ER-dimer is translocated into the nucleus and activates recruitment of transcriptional coactivators via binding to the ERE. This leads to transcriptional activation (indicated by a black arrow) and breast cell growth.

References

1. McDonagh EM, Whirl-Carrillo M, Garten Y, Altman RB, Klein TE (2011) From pharmacogenomic knowledge acquisition to clinical applications: the PharmGKB as a clinical pharmacogenomic biomarker resource. Biomarkers in Medicine 5: 795-806.
2. Rae JM (2011) Personalized tamoxifen: what is the best way forward? Journal of clinical oncology : official journal of the American Society of Clinical Oncology 29: 3206-3208.
