# Supplementary material for: CYP2D6 Genotype and Tamoxifen Response for Breast Cancer: A Systematic Review and Meta-Analysis
Source: PLoS One. 2013 Oct 2;8(10):e76648. doi: 10.1371/journal.pone.0076648 (PMC3788742; doi:10.1371/journal.pone.0076648)
Supplement: Figure S2 — Generation of the composite outcomes from outcomes reported in the studies identified from the systematic review. (PDF) [file pone.0076648.s010.pdf]

Figure S2: Generation of the composite outcomes from outcomes reported in the studies identified from the systematic review.

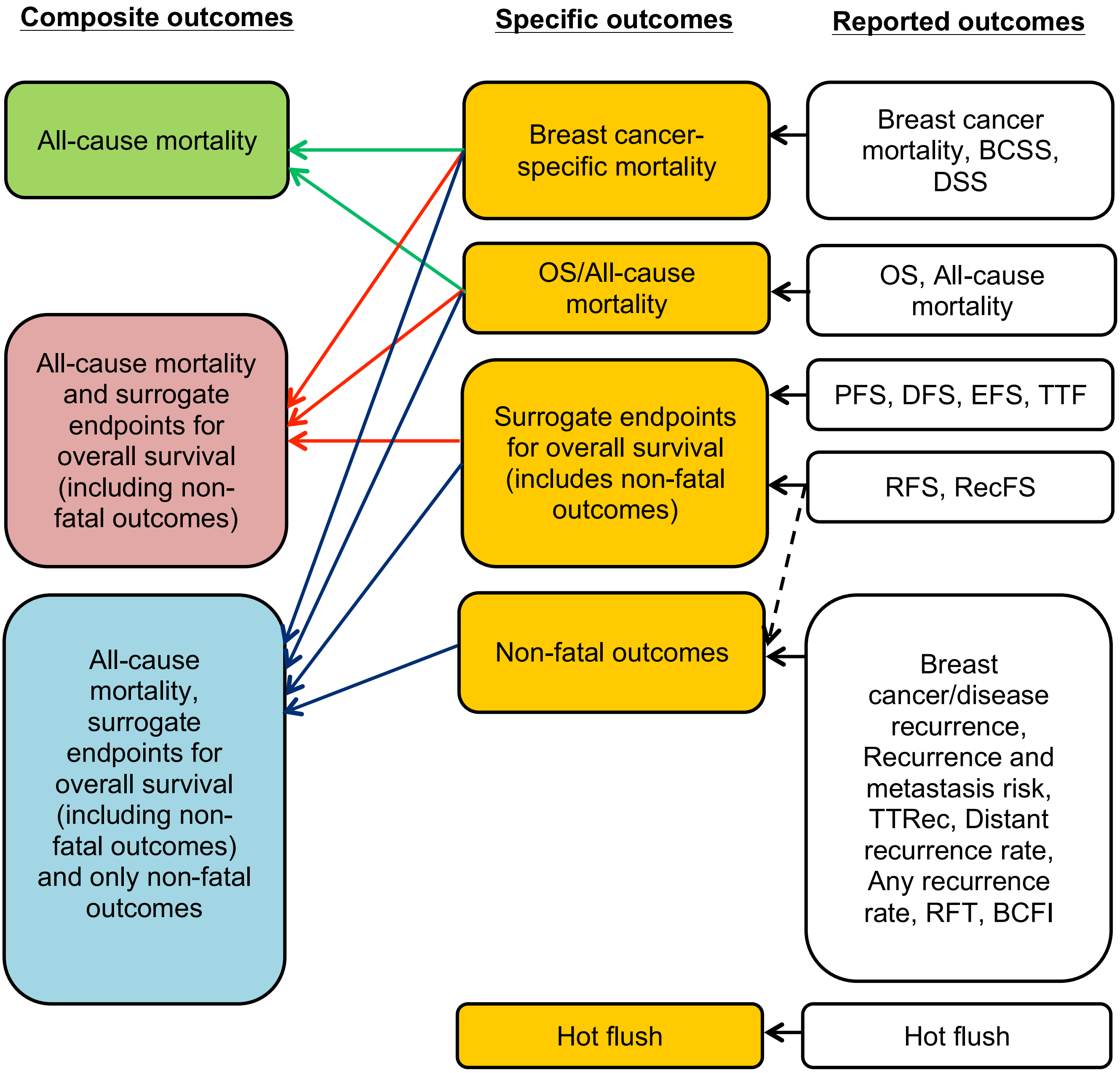

Footnotes: BCFI: breast cancer-free interval, BCSS: breast cancer-specific survival, DFS: disease-free survival, DSS: disease-specific survival, EFS: event-free survival, OS: overall survival, PFS: progression-free survival, RFS: relapse-free survival, RFT: relapse-free time RecFS: recurrence-free survival, TTF: time to-treatment failure, TTRec: time to recurrence. Dash arrow: reported outcomes included only for specific outcome meta-analysis.
