# Supplementary material for: CYP2D6 Genotype and Tamoxifen Response for Breast Cancer: A Systematic Review and Meta-Analysis
Source: PLoS One. 2013 Oct 2;8(10):e76648. doi: 10.1371/journal.pone.0076648 (PMC3788742; doi:10.1371/journal.pone.0076648)
Supplement: Figure S3 — PRISMA flow diagram for systematic review of the association of CYP2D6 genotype and tamoxifen response in breast cancer patients. (PDF) [file pone.0076648.s011.pdf]

Figure S3: PRISMA flow diagram for systematic review of the association of *CYP2D6* genotype and tamoxifen response in breast cancer patients.

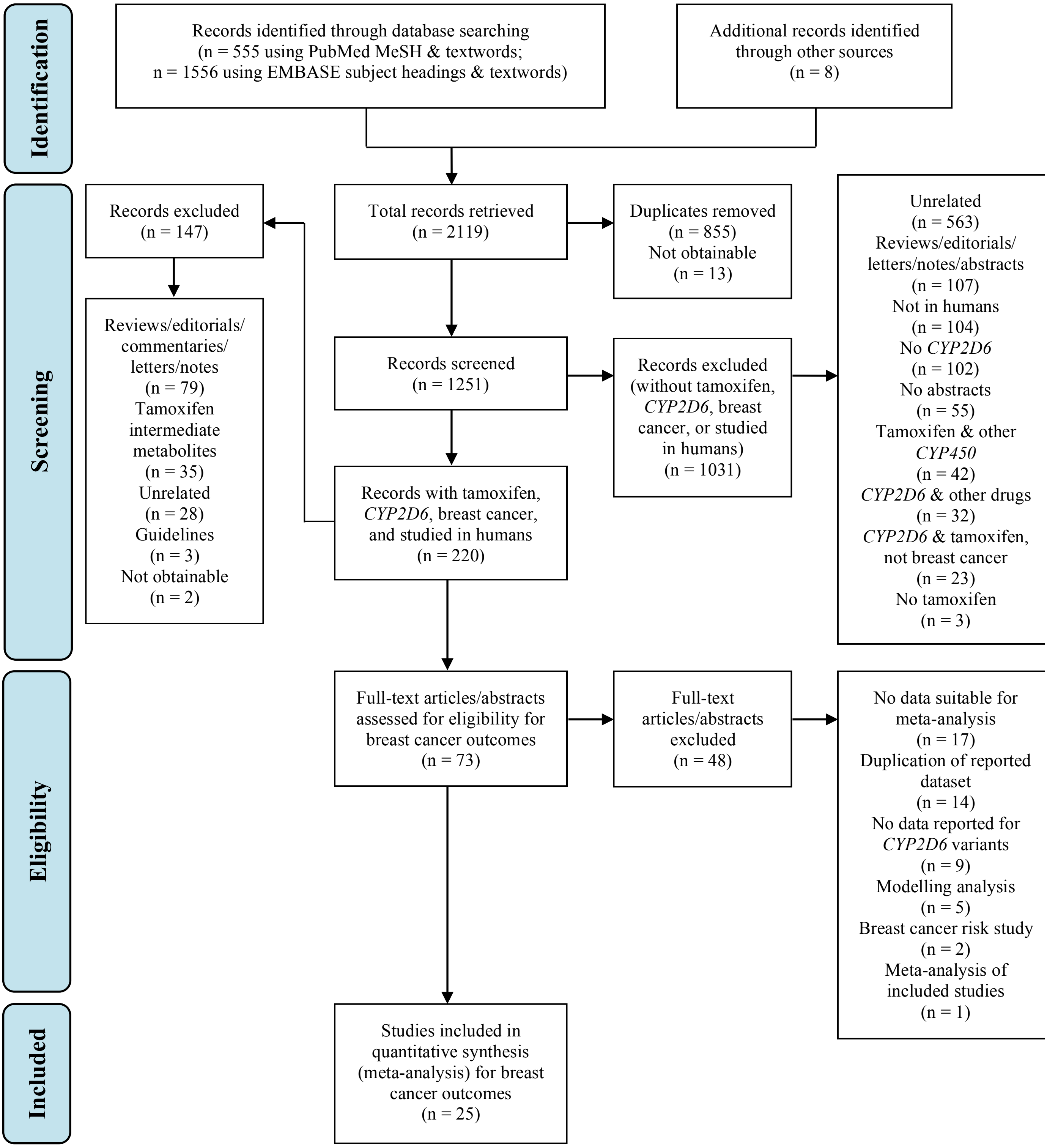

Footnotes: The flow diagram template was adopted from the PRISMA statement.[1]

References

1. Moher D, Liberati A, Tetzlaff J, Altman DG (2009) Preferred reporting items for systematic reviews and meta-analyses: the PRISMA statement. PLoS Medicine 6: e1000097.
