# Supplementary material for: CYP2D6 Genotype and Tamoxifen Response for Breast Cancer: A Systematic Review and Meta-Analysis
Source: PLoS One. 2013 Oct 2;8(10):e76648. doi: 10.1371/journal.pone.0076648 (PMC3788742; doi:10.1371/journal.pone.0076648)
Supplement: Figure S4 — Unique outcomes reported by studies and the grouping into main outcomes for analyses. (PDF) [file pone.0076648.s012.pdf]

Figure S4: Unique outcomes reported by studies and the grouping into main outcomes for analyses.

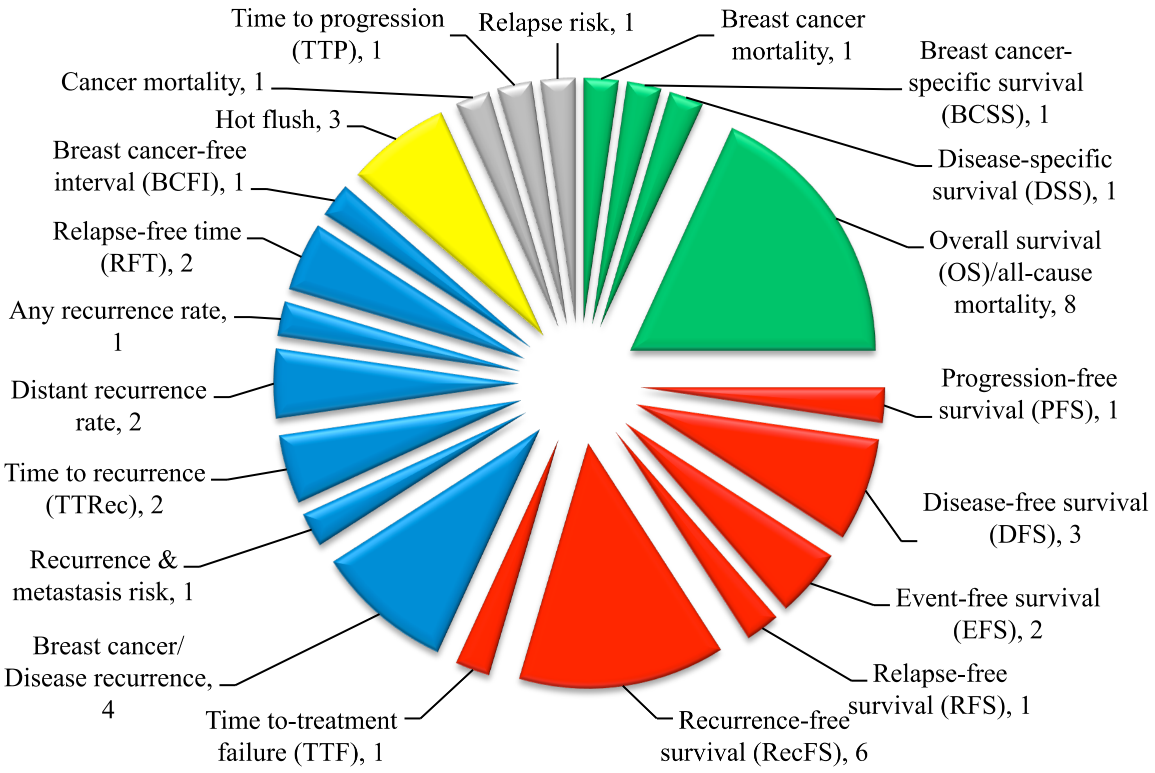

Footnotes: The number indicates the number of studies that reported each outcome. The segments are color coded according to which outcome they were grouped into: (i) green for outcomes (n=11) that were grouped into all-cause mortality; (ii) red for outcomes (n=14) that were grouped into surrogate endpoints for overall survival (including non-fatal events); (iii) blue for outcomes (n=13) that were grouped into non-fatal events only. The segment colored yellow shows outcomes (n=3) that represented adverse drug reactions. The grey segment represents outcomes (n=3) that were unsuitable to be included for analyses on the basis of their unclear definitions.
