# Supplementary material for: CYP2D6 Genotype and Tamoxifen Response for Breast Cancer: A Systematic Review and Meta-Analysis
Source: PLoS One. 2013 Oct 2;8(10):e76648. doi: 10.1371/journal.pone.0076648 (PMC3788742; doi:10.1371/journal.pone.0076648)
Supplement: Figure S5 — Proportions of 13,629 individuals in the 25 studies that were genotyped for the 36 CYP2D6 * alleles. (PDF) [file pone.0076648.s013.pdf]

Figure S5: Proportions of 13,629 individuals in the 25 studies that were genotyped for the 36 *CYP2D6* * alleles.

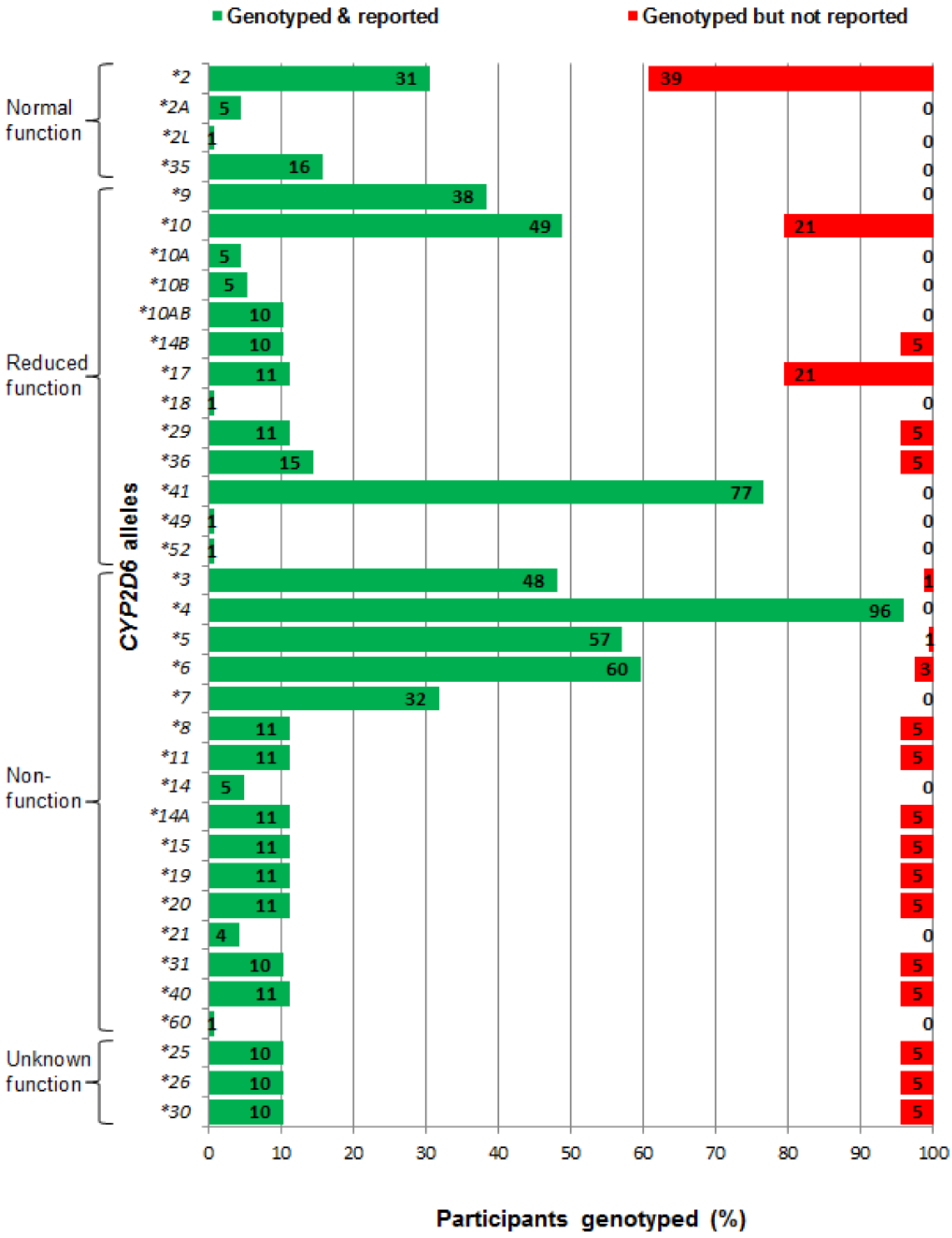

Footnotes: *CYP2D6* alleles genotyped and reported are represented by the green bars, and *CYP2D6* alleles genotyped but not reported are represented by the red bars. The number inside the bars represents the percentage of the total participants genotyped. The Y-axis shows the function of the *CYP2D6* alleles referenced from the *CYP2D6* allele nomenclature[1] and from the recent updated sequencing data for the *CYP2D6*11* allele,[2] or suggested from the original articles linked to from the *CYP2D6* allele nomenclature website (http://www.cypalleles.ki.se) if no functional information was available.

References

1. Sim SC (2012) CYP2D6 allele nomenclature.

2. Skierka JM, Walker DL, Peterson SE, O'Kane DJ, Black JL (2012) CYP2D6*11 and challenges in clinical genotyping of the highly polymorphic CYP2D6 gene. Pharmacogenomics 13: 951-954.
