# Supplementary material for: CYP2D6 Genotype and Tamoxifen Response for Breast Cancer: A Systematic Review and Meta-Analysis
Source: PLoS One. 2013 Oct 2;8(10):e76648. doi: 10.1371/journal.pone.0076648 (PMC3788742; doi:10.1371/journal.pone.0076648)
Supplement: Figure S6 — Meta-analysis (fixed and random effects models) of the association of any reduced function CYP2D6 allele versus none for the risk of all-cause mortality. (PDF) [file pone.0076648.s014.pdf]

**Figure S6: Meta-analysis (fixed and random effects models) of the association of any reduced function *CYP2D6* allele versus none for the risk of all-cause mortality.**

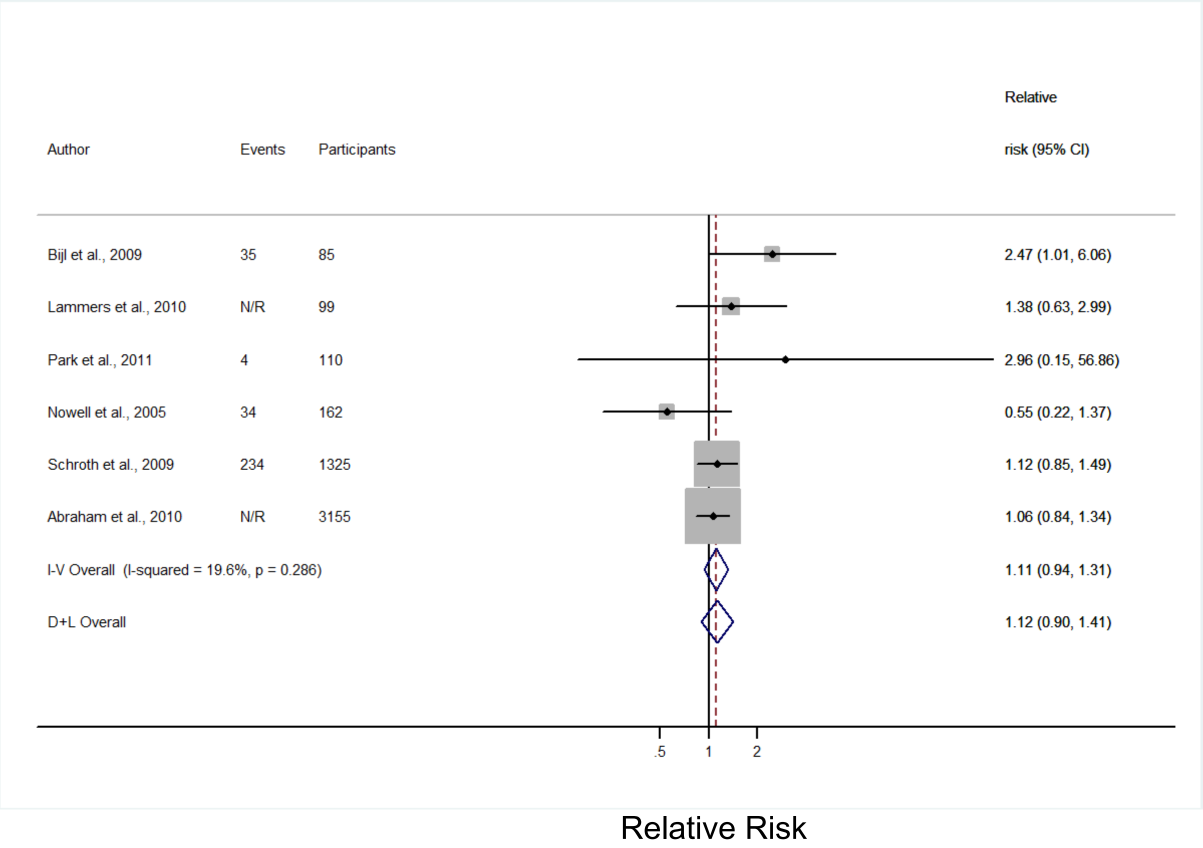


**Footnotes**: Relative risk (RR) and the 95% confidence intervals (CI) for each study are represented by the black diamond and the width of the horizontal line respectively. The weight for each study is ascertained under the fixed effects model and is represented by the grey squares, with a larger square area indicating heavier weighting. The diamond represents the overall effect and the red vertical dash line represents the overall RR (for fixed effects model) and the diamond's width represents the 95% CI for the overall RR. Overall heterogeneity among the studies is reported as I-squared ($I^2$), tested using using Cochrane's Q P-value. Fixed effects model is reported as I-V (inverse variance) and random effects model is reported as D+L (DerSimonian and Laird). N/R: not reported.
