# Supplementary material for: CYP2D6 Genotype and Tamoxifen Response for Breast Cancer: A Systematic Review and Meta-Analysis
Source: PLoS One. 2013 Oct 2;8(10):e76648. doi: 10.1371/journal.pone.0076648 (PMC3788742; doi:10.1371/journal.pone.0076648)
Supplement: Figure S8 — Meta-analysis (fixed and random effects models) of the association of any reduced function CYP2D6 allele versus none with the composite outcome of all-cause mortality, surrogate endpoints for overall survival (including non-fatal events) and non-fatal events. (PDF) [file pone.0076648.s016.pdf]

Figure S8: Meta-analysis (fixed and random effects models) of the association of any reduced function *CYP2D6* allele versus none with the composite outcome of all-cause mortality, surrogate endpoints for overall survival (including non-fatal events) and non-fatal events.

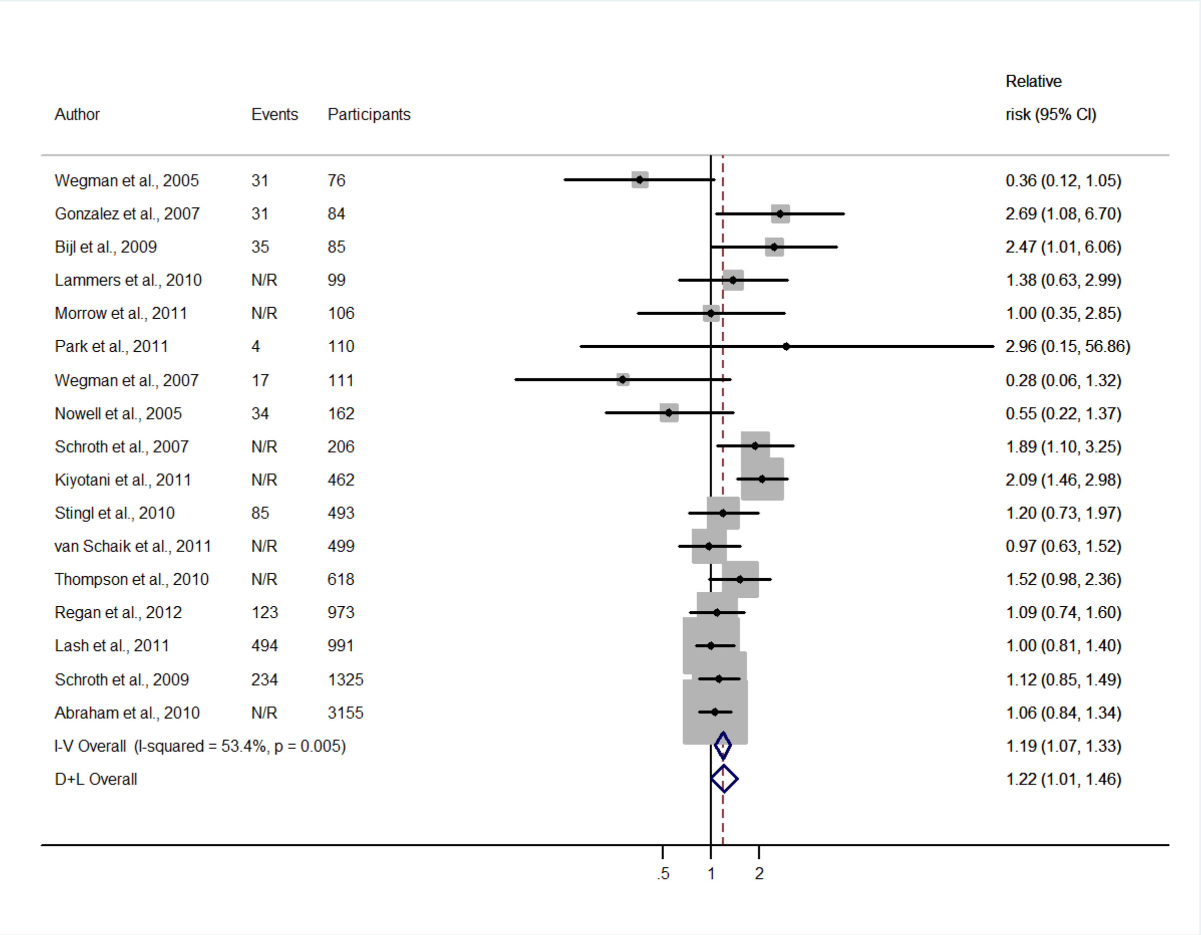

Relative Risk

Footnotes: Fixed effects model is reported as I-V (inverse variance) and random effects model is reported as D+L (DerSimonian and Laird). N/R: not reported.
