# Supplementary material for: CYP2D6 Genotype and Tamoxifen Response for Breast Cancer: A Systematic Review and Meta-Analysis
Source: PLoS One. 2013 Oct 2;8(10):e76648. doi: 10.1371/journal.pone.0076648 (PMC3788742; doi:10.1371/journal.pone.0076648)
Supplement: Figure S9 — Subgroup analysis of the association of any reduced function CYP2D6 allele versus none for the composite outcome of all-cause mortality and surrogate endpoints for overall survival (including non-fatal events). (PDF) [file pone.0076648.s017.pdf]

Figure S9: Subgroup analysis of the association of any reduced function *CYP2D6* allele versus none for the composite outcome of all-cause mortality and surrogate endpoints for overall survival (including non-fatal events).

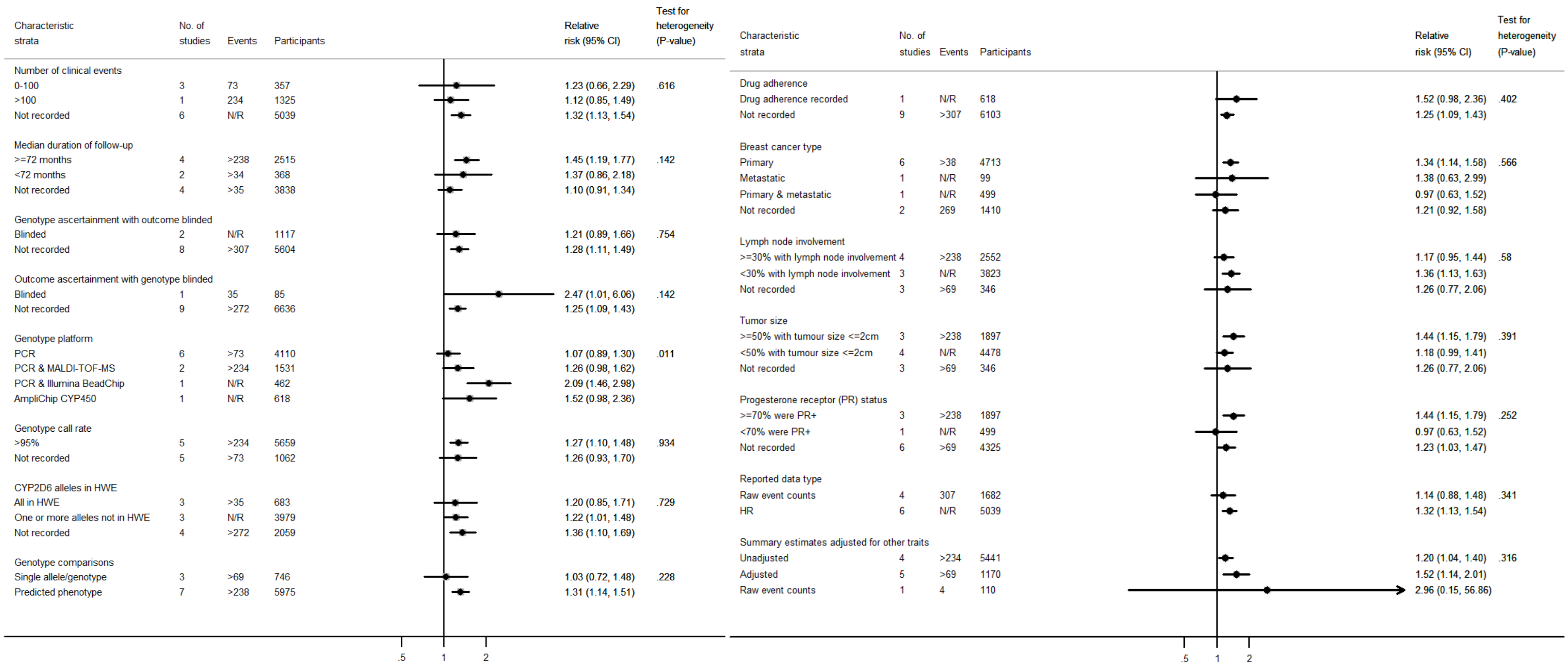

Characteristic strata	No. of studies	Events	Participants	Relative risk (95% CI)	Test for heterogeneity (P-value)
Number of clinical events					
0-100	3	73	357	1.23 (0.66, 2.29)	.616
>100	1	234	1325	1.12 (0.85, 1.49)	
Not recorded	6	N/R	5039	1.32 (1.13, 1.54)	
Median duration of follow-up					
>=72 months	4	>238	2515	1.45 (1.19, 1.77)	.142
<72 months	2	>34	368	1.37 (0.86, 2.18)	
Not recorded	4	>35	3838	1.10 (0.91, 1.34)	
Genotype ascertainment with outcome blinded					
Blinded	2	N/R	1117	1.21 (0.89, 1.66)	.754
Not recorded	8	>307	5604	1.28 (1.11, 1.49)	
Outcome ascertainment with genotype blinded					
Blinded	1	35	85	2.47 (1.01, 6.06)	.142
Not recorded	9	>272	6636	1.25 (1.09, 1.43)	
Genotype platform					
PCR	6	>73	4110	1.07 (0.89, 1.30)	.011
PCR & MALDI-TOF-MS	2	>234	1531	1.26 (0.98, 1.62)	
PCR & Illumina BeadChip	1	N/R	462	2.09 (1.46, 2.98)	
AmpliChip CYP450	1	N/R	618	1.52 (0.98, 2.36)	
Genotype call rate					
>95%	5	>234	5659	1.27 (1.10, 1.48)	.934
Not recorded	5	>73	1062	1.26 (0.93, 1.70)	
CYP2D6 alleles in HWE					
All in HWE	3	>35	683	1.20 (0.85, 1.71)	.729
One or more alleles not in HWE	3	N/R	3979	1.22 (1.01, 1.48)	
Not recorded	4	>272	2059	1.36 (1.10, 1.69)	
Genotype comparisons					
Single allele/genotype	3	>69	746	1.03 (0.72, 1.48)	.228
Predicted phenotype	7	>238	5975	1.31 (1.14, 1.51)	

Characteristic strata	No. of studies	Events	Participants	Relative risk (95% CI)	Test for heterogeneity (P-value)
Drug adherence					
Drug adherence recorded	1	N/R	618	1.52 (0.98, 2.36)	.402
Not recorded	9	>307	6103	1.25 (1.09, 1.43)	
Breast cancer type					
Primary	6	>38	4713	1.34 (1.14, 1.58)	.566
Metastatic	1	N/R	99	1.38 (0.63, 2.99)	
Primary & metastatic	1	N/R	499	0.97 (0.63, 1.52)	
Not recorded	2	269	1410	1.21 (0.92, 1.58)	
Lymph node involvement					
>=30% with lymph node involvement	4	>238	2552	1.17 (0.95, 1.44)	.58
<30% with lymph node involvement	3	N/R	3823	1.36 (1.13, 1.63)	
Not recorded	3	>69	346	1.26 (0.77, 2.06)	
Tumor size					
>=50% with tumour size <=2cm	3	>238	1897	1.44 (1.15, 1.79)	.391
<50% with tumour size <=2cm	4	N/R	4478	1.18 (0.99, 1.41)	
Not recorded	3	>69	346	1.26 (0.77, 2.06)	
Progesterone receptor (PR) status					
>=70% were PR+	3	>238	1897	1.44 (1.15, 1.79)	.252
<70% were PR+	1	N/R	499	0.97 (0.63, 1.52)	
Not recorded	6	>69	4325	1.23 (1.03, 1.47)	
Reported data type					
Raw event counts	4	307	1682	1.14 (0.88, 1.48)	.341
HR	6	N/R	5039	1.32 (1.13, 1.54)	
Summary estimates adjusted for other traits					
Unadjusted	4	>234	5441	1.20 (1.04, 1.40)	.316
Adjusted	5	>69	1170	1.52 (1.14, 2.01)	
Raw event counts	1	4	110	2.96 (0.15, 56.86)	

Footnotes: CI: confidence interval, CYP450: cytochrome P450, HR: hazard ratio, HWE: Hardy-Weinberg equilibrium, MALDI-TOF-MS: matrix-assisted laser desorption/ionization time-of-flight mass spectrometry, N/R: not recorded, PCR: polymerase chain reaction.
