# Supplementary material for: CYP2D6 Genotype and Tamoxifen Response for Breast Cancer: A Systematic Review and Meta-Analysis
Source: PLoS One. 2013 Oct 2;8(10):e76648. doi: 10.1371/journal.pone.0076648 (PMC3788742; doi:10.1371/journal.pone.0076648)

Figure S11: Influence analysis of any reduced function *CYP2D6* allele versus none for the composite outcomes.

All-cause mortality

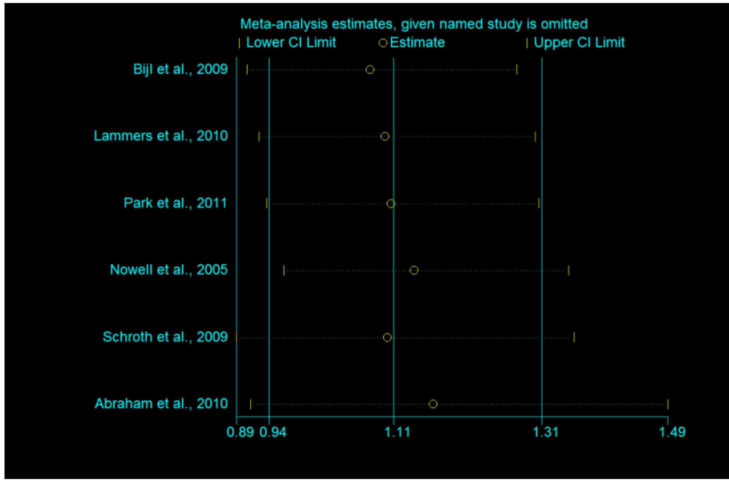

All-cause mortality and surrogate endpoints for overall survival (including non-fatal events)

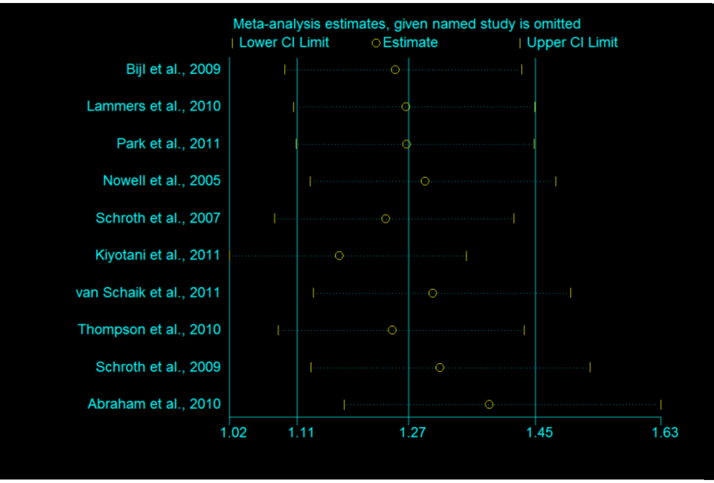

All-cause mortality, surrogate endpoints for overall survival (including non-fatal events) and non-fatal outcomes

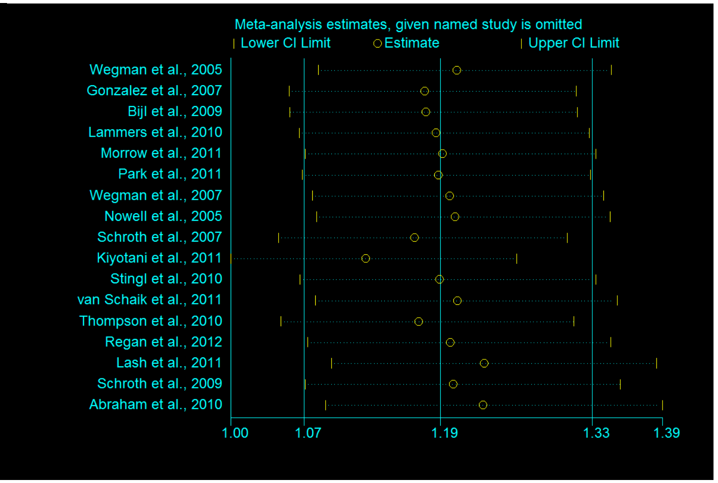

Supplement: Figure S11 — Influence analysis of any reduced function CYP2D6 allele versus none for the composite outcomes. (PDF) [file pone.0076648.s019.pdf]
